# *Andrographis paniculata* (Chuān Xīn Lián) for symptomatic relief of acute respiratory tract infections in adults and children: A systematic review and meta-analysis

**DOI:** 10.1371/journal.pone.0181780

**Published:** 2017-08-04

**Authors:** Xiao-Yang Hu, Ruo-Han Wu, Martin Logue, Clara Blondel, Lily Yuen Wan Lai, Beth Stuart, Andrew Flower, Yu-Tong Fei, Michael Moore, Jonathan Shepherd, Jian-Ping Liu, George Lewith

**Affiliations:** 1 Primary Care and Population Sciences, Aldermoor Health Centre, Southampton, United Kingdom; 2 Centre for Evidence-Based Chinese Medicine, Beijing University of Chinese Medicine, Beijing, China; 3 AgroParisTech, Paris Institute of Technology for Life, Food and Environmental Sciences, Paris, France; 4 Southampton Health Technology Assessments Centre (SHTAC), Faculty of Medicine, University of Southampton, Southampton, United Kingdom; Defence Research and Development Organisation, INDIA

## Abstract

**Introduction:**

Antimicrobial resistance (AMR) is a substantial threat to public health. Safe and effective alternatives are required to reduce unnecessary antibiotic prescribing. *Andrographis Paniculata* (*A*. *Paniculata*, Chuān Xīn Lián) has traditionally been used in Indian and Chinese herbal medicine for cough, cold and influenza, suggesting a role in respiratory tract infections (RTIs). This systematic review aimed to evaluate the clinical effectiveness and safety of *A*. *Paniculata* for symptoms of acute RTIs (ARTIs).

**Materials and methods:**

English and Chinese databases were searched from their inception to March 2016 for randomised controlled trials (RCTs) evaluating oral *A*. *Paniculata* without language barriers (Protocol ID: CRD42016035679). The primary outcomes were improvement in ARTI symptoms and adverse events (AEs). A random effects model was used to pool the mean differences and risk ratio with 95% CI reported. Methodological quality was evaluated using the Cochrane risk of bias tool; two reviewers independently screened eligibility and extracted data.

**Results:**

Thirty-three RCTs (7175 patients) were included. Most trials evaluated *A*. *Paniculata* (as a monotherapy and as a herbal mixture) provided commercially but seldom reported manufacturing or quality control details. *A*. *Paniculata* improved cough (n = 596, standardised mean difference SMD: -0.39, 95% confidence interval CI [-0.67, -0.10]) and sore throat (n = 314, SMD: -1.13, 95% CI [-1.37, -0.89]) when compared with placebo. *A*. *Paniculata* (alone or plus usual care) has a statistically significant effect in improving overall symptoms of ARTIs when compared to placebo, usual care, and other herbal therapies. Evidence also suggested that *A*. *Paniculata* (alone or plus usual care) shortened the duration of cough, sore throat and sick leave/time to resolution when compared versus usual care. No major AEs were reported and minor AEs were mainly gastrointestinal. The methodological quality of included trials was overall poor.

**Conclusions:**

*A*. *Paniculata* appears beneficial and safe for relieving ARTI symptoms and shortening time to symptom resolution. However, these findings should be interpreted cautiously owing to poor study quality and heterogeneity. Well-designed trials evaluating the effectiveness and potential to reduce antibiotic use of *A*. *Paniculata* are warranted.

## Introduction

Respiratory tract infections (RTIs) are one of the most common reason for primary care consultations in the UK [1]. Treatments for RTIs are mainly symptomatic [2], and often include analgesics, antipyretics [3], mucolytics, expectorants, decongestants [4], and educational interventions [5], although evidence supporting currently used symptomatic treatment is still limited [6]. Antibiotics are frequently prescribed in primary care settings in Europe [7] with 60% of all antibiotic prescribing in the UK occurring in primary care [1]. Research has suggested RTIs are predominantly of viral aetiology [8], and that antibiotics are of very limited benefit in the majority of uncomplicated infections [9, 10]. Systematic reviews to date have failed to provide evidence for the effectiveness of antibiotics for RTIs [11]. Antibiotics showed no benefit in symptom improvement for acute RTIs (ARTIs) such as colds [12], persisting acute purulent rhinitis [12], or acute laryngitis [13]; and suggested little absolute benefits for reducing symptom duration or complications in sore throat [14], bronchitis [15, 16], sinusitis [17] and acute otitis media [18].

Antimicrobial resistance (AMR) is an evolving major global threat to public health [19]. A recent Public Health England report showed a 6% increase in total antibiotic use in England between 2010 and 2013 and it remains an important government priority to reduce antibiotic prescribing [20, 21]. The marginal benefit of antibiotics for ARTIs are outweighed by increasing AMR and common adverse reactions [3] leading to unnecessary increases in healthcare costs [22–24].

Research is urgently needed to explore other treatments that may be offered for symptomatic relief to reduce unnecessary antibiotic prescribing. In order to facilitate rapid translation of research into clinical practice, there has been much interest in researching options currently available to the general public. This has involved over the counter (OTC) pharmacological treatments such as paracetamol as well as herbal alternatives. Evidence from previous systematic reviews suggested promising but limited evidence for Chinese herbs in influenza [25], common colds [26], upper RTI [27], and cough [28].

*A*. *Paniculata* (Burm.f.) Wall ex Nees (Acanthaceae family), also known as nemone chinensi, Chuān Xīn Lián, has traditionally been used in Indian and Chinese herbal medicine. It is traditionally used as an antipyretic for relieving and reducing the severity and duration of symptoms of common colds and alleviating fever, cough and sore throats, or as a tonic to aid convalescence after uncomplicated RTIs [29][30]. There is encouraging evidence to demonstrate the potential mechanistic for effects of *A*. *Paniculata* for RTIs. The active constituents of *A*. *Paniculata* include the diterpene, lactones commonly known as the andrographolides which have shown anti-inflammatory, antiviral, anti-allergic, and immune-stimulatory activities [31]. They inhibit platelet-activating factor mediated inflammatory response [32], reduces expression of pro-inflammatory proteins such as cyclooxygenase-2 [33, 34], and demonstrates analgesic effects as well as antipyretic effects comparable to paracetamol [35]. *A*. *Paniculata* has also been shown, in vitro, to be effective against avian influenza A (H9N2 and H5N1) and human influenza A H1N1 viruses, possibly through blocking the binding of viral hemagglutinin to cells [36], or by inhibiting H1N1 virus-induced cell death [37].

Two previous systematic reviews showed that *A*. *Paniculata* alone or in combination with *A*. *senticosus* is superior to placebo for reducing symptom severity in upper RTIs [38, 39]. However, the clinical evidence for *A*. *Paniculata* for symptoms of lower RTI has not yet been systematically evaluated and would be important to review prior to conducting further research in this area. Furthermore, previous systematic reviews have been limited to English-languages searches and given that *A*. *Paniculata* is used in Indian and Chinese herbal medicine, an up-to-date systematic review without language restrictions is warranted.

This systematic review therefore evaluated the clinical efficacy, effectiveness and safety of *A*. *Paniculata* for of the treatment of ARTIs.

## Materials and methods

This systematic review followed PRISMA reporting guidelines (S1 Table). A protocol of this review has been registered (CDR: CRD42016035679, S1 File). Ethics statement: N/A.

### Search strategy and study selection

MEDLINE, EMBASE, AMED, Cochrane Library, CINAHL, China National Knowledge Infrastructure (CNKI), Wan Fang, Sino-Med Database, and Chinese Science and Technology Journal Database (VIP) were searched from their inception to March 2016. A range of freetext words and indexed terms related to “*Andrographis Paniculata*” and “respiratory tract infection” were searched. The reference lists of studies meeting the inclusion criteria were searched to identify additional relevant studies. A detailed search strategy and search term alternatives for each database are available as supporting information; see S2 File. There were no exclusions made based on language. Literature searching (XYH, RHW) was followed by independently screening with at least two authors (XYH, RHW, ML). Study authors were contacted to obtain relevant missing data if necessary and where resources allowed.

### Data extraction and management

A data extraction spreadsheet was designed and piloted with appropriate changes made for this review. The form identified trial characteristics, characteristics of trial population and conditions, details of interventions in all trial arms according to the consolidated standards of reporting trials (CONSORT) herbal extension in terms of features of herbal intervention [40], details of concomitant interventions, quality assessment, and findings on efficacy, effectiveness and AEs. Two reviewers extracted study data independently for Chinese-language (XYH, RHW, LL) and English-language (ML, CB) trials, with findings compared and agreed.

### Eligibility criteria

This review included published and unpublished randomised controlled trials (RCTs). Quasi-RCTs, crossover trials, controlled before and after studies, interrupted time series (ITS) studies, and non-experimental studies were not included due to their potential high risk of bias.

Studies of human participants of all ages, with symptoms of ARTIs. A clinical diagnosis of ARTI was the main inclusion criteria. Diagnoses of upper or lower ARTIs include acute common cold, influenza, rhinosinusitis, laryngitis, tonsillitis, pharyngitis, croup, acute otitis media, bronchitis, pneumonia, and acute exacerbations of chronic obstructive pulmonary disease (COPD). Symptoms of ARTIs are defined as having symptoms such as cough, sore throat, fever, runny nose and discoloured sputum for a duration of less than four weeks. Trials were excluded if they recruited participants with asthma, had active or previous peptic ulceration, were hypersensitive to analgesics, had psychosis, or were severely depressed. Exclusion also applied to trials that included patients who required hospital admission (for example, for meningitis, severe pneumonia, epiglottitis, or Kawasaki disease), had a known immune deficiency, or were pregnant or breastfeeding [41].

Examples of herbal mixture include: products containing *A*. *Paniculata* in combination with Scutellaria baicalensis, or in combination with Lonicera japonica, Forsythia suspense, and Aster trinervius. No limitation was imposed concerning dosage, methods of dosing or duration of administration.

We included comparisons such as placebo or no intervention; usual care such as analgesics, antivirals, antibiotics, anti-inflammatories, steroids or corticosteroids; or other herbal remedies. Studies comparing different preparations of *A*. *paniculata*, e.g. comparing tablet with granule, were also included in this review.

### Outcome measures

The following primary outcome measures were included in this review:

1. Participant self-reported or clinician/observer assessment on overall ARTI symptoms; or two target symptoms cough and sore throat. Commonly used measures included:
  - Changes on visual analogue scales (VAS)
  - Changes in symptoms scored on a Likert-type scale
  - Global assessment of symptom improvement by the patient
  - Global assessment of symptom improvement by treating clinician
2. AEs: This included any anaphylactic, allergic reactions, hypersensitivity reactions, or complications of *A*. *Paniculata*, such as rash, nausea, fatigue, or worsening of ARTIs symptoms. We also collected information regarding AEs due to interactions among *A*. *Paniculata* in combination with other remedies, or potential interactions with medications patients had for their co-morbidities.

We defined serious AEs according to the International Council on Harmonisation of Technical Requirements for Registration of Pharmaceuticals for Human Use (ICH) guidelines as any event that leads to death, is life-threatening, requires hospitalisation or leads to persistent or significant disability; biochemistry results such as electrolytes, liver and kidney function tests (alanine aminotransferase and creatinine) [42].

Secondary outcome measures included:

1. Mean time to reported remission or resolution of symptoms. This may be measured directly, through patient or clinician/observer report or indirectly as the time to return to normal activities.
2. Reduction in reported antibiotic usage, e.g. number of scripts issued immediately at the time of consultation and uptake of delayed prescriptions. Although the Chinese government launched a special campaign to promote the rational use of antimicrobials in healthcare settings in the 2011 healthcare reform, this has yet to be implemented in many places in China [43]. Antibiotics are prescribed on patients’ initial visit if there were suspicions of bacteria inflammation, therefore scripts immediately issued at the time of consultation was recorded.

Trials that did not report either our primary and or secondary outcome measures were excluded from this review.

Timing of effect measures: Some studies may have used a repeated measures approach. Timings of measures for each included trial were documented with commonly reported time points explored if there was sufficient data available. All outcome measures were assessed at baseline and data for all time points were extracted with the aim to pooling those trials that collected data at similar time points. Otherwise, data at the most appropriate follow-up point were assessed.

### Assessment of risk of bias in included studies

The risk of bias of the included RCTs was assessed independently by two reviewers using the tool developed by Higgins and Green in the Cochrane Handbook for Systematic Reviews of Interventions [44]. We assessed bias over the following domains: selection bias (random sequence generation and allocation concealment), performance bias (blinding of participants and personnel), detection bias (blinding of researchers conducting outcome assessments), attrition bias (incomplete outcome data), reporting bias (selective reporting), and other sources of bias. A judgement of ‘low risk’ of bias, ‘high risk’ or bias, or ‘unclear risk’ of bias was provided for each domain. Any disagreements were resolved by discussion or by involving a third reviewer until consensus was reached.

### Measures of treatment effect

Data from individual studies were combined in a meta-analysis when interventions were performed in a homogeneous clinical environment, with similar population, settings, intervention and comparison, and outcome measures. Overall effect sizes were estimated using Review Manager (RevMan) Version [5.3] [45]. Copenhagen: The Nordic Cochrane Centre, The Cochrane Collaboration, 2014. Because of the anticipated variability in the populations and interventions of included trials, a generic inverse variance random effects model was used to pool the mean difference (MD) with 95% confidence interval (CI) on target continuous outcomes to incorporate heterogeneity [46, 47]. When the units of the outcome measures used across studies were not consistent, the effects as standardised mean differences (SMD) were reported. An overall effect size of 0.2–0.5 was regarded as small, 0.5–0.8 as moderate and more than 0.8 as large [48]. For dichotomous data, a random effects method was used to pool the summary risk ratio (RR) with 95% CI. Absolute risk estimates were calculated using the event rates of control groups as baseline risks.

### Dealing with missing data

Where data was missing or incomplete, we contacted study authors to obtain this where possible. If the means were reported without standard deviations, we calculated the standard deviation from the information reported such as p-values, F-values or confidence intervals. As far as possible, we utilised intention to treat (ITT) analysis data for all outcomes. However, most included trials reported complete cases only; and complete case data were the primary analysis dataset. For each outcome, the number of participants whose data was available at baseline and at follow up, and the rate of loss to follow-up were recorded.

### Assessment of heterogeneity

Between-study heterogeneity was assessed using the I^2^- statistic which describes the percentage of variation across studies that is due to heterogeneity rather than chance. Rules of thumb for interpretation of this statistic suggest that I^2^>30% equates to moderate heterogeneity, I^2^>50% equates to substantial heterogeneity and I^2^>75% equates to considerable heterogeneity [46]. For all I^2^ values above 50%, we investigated potential sources of heterogeneity. Although this threshold is widely used, it is somewhat arbitrary and therefore if the I^2^ value was below 50% but the direction and magnitude of treatment effects suggest important heterogeneity, we investigated the potential sources in a sensitivity analysis and took this into account when interpreting the findings. As high levels of heterogeneity were expected due to complexity in the form of *A*. *Paniculata* (e.g. monotherapy or herbal mixture, capsule or liquid), it was planned to use a random effects model to pool the overall effects [46].

### Assessment of reporting biases

Funnel plots were created to investigate potential reporting bias where this was feasible and there were sufficient studies [49]. Funnel plot tests for asymmetry were conducted separately in STATA software version 14 using the metabias command.

### Sensitivity analysis

Sensitivity analyses were conducted for the primary outcomes to determine whether the review conclusions would have differed if eligibility was restricted to trials without high or unclear risk of bias for either in sequence generation or allocation concealment domains) [46]; and if eligibility was restricted to trials that provided any detail on authentication or standardisation of the herb.

### Subgroup analysis

If there was sufficient available data, several subgroup analyses were planned *a priori* to compare the effect estimate between studies that evaluated:

- Patients with upper ARTIs versus lower ARTIs;
- Adults versus children (younger than 18);
- *A*. *Paniculata* as monotherapy versus as fixed combinations;
- *A*. *Paniculata* in different preparation, e.g. granule versus tablet or other forms

## Results

### Description of included trials

**Fig 1:**
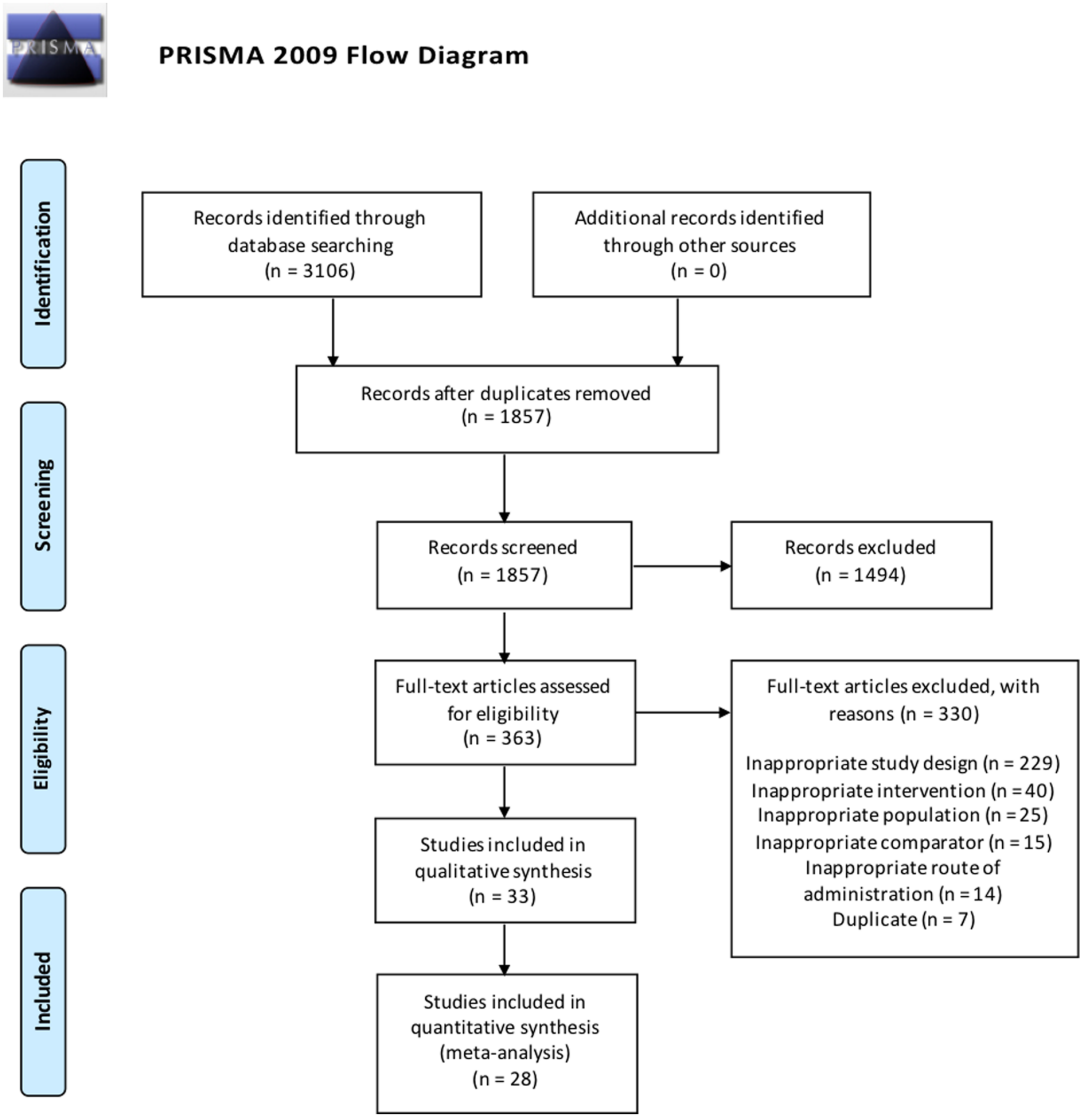
Flow and identification of trials to include in review.

The literature search identified 3106 studies, of which a final total of 33 RCTs [50–82], comprising 7175 patients, met the criteria to be included (Fig 1). Authors of two trials [52, 69] were contacted for further information but received no response. Tables 1–5 shows the characteristics of the 33 included trials. The included trials were published between 1991 and 2014, with 25 from China [50, 51, 53–58, 60–68, 71–75, 80–82], three from Russia [59, 70, 79], two from Sweden [77, 79], and one each from Thailand [52], India [78], and Chile [76]. Two were three-armed trials [52, 70], and the remaining were two-armed parallel RCTs [50, 51, 53–69, 71–82].

**Table 1:** Trial characteristics: *A*. *Paniculata* versus Placebo (n = 4).

STUDY ID	Diagnosis (syndrome differentiation)	Course of symptoms: mean±SD	Age: Mean±SD (y)	Gender (% of male)	N (analysed/recruited)	Name of the TG product & co-intervention if available	Details of CG	Outcome measures	End point

Caceres et al., 1999 [76] Chile	Common cold	NR	NR; 25–50 as inclusion criteria	TG: 53.9%; CG: 45.2%	158/208	AP mono (tablet)	Placebo tablet, 4 tablets, tid, 5d	[ITT] Improvement in cough intensity and frequency (VAS, 10cm)	0–4
Melchior et al., 1997 [77] Sweden	Common cold	Within 3d	NR	NR	50/50	AP mono (tablet)	Placebo tablet, 400mg, tid, 5d	CCME (patient reported); Symptom relief (VAS)	5
Saxena et al., 2010 [78] India	Uncomplicated URTIs	Within 3d	TG: 34.36±0.97; CG: 32.42±1.1	TG: 67%; CG: 62%	220/223	AP mono (capsule)	Placebo capsules, 300mg, bid, 5d	[PP data] Severity of overall severity of 8 symptoms (VAS, 0–100); Severity of cough (VAS, 0–100); Severity of sore throat (VAS, 0–100)	5
Melchior et al., 2000 [79] Russia	Uncomplicated URTIs	Within 36h	Range: 18–55 (inclusion criteria)	NR	178/179	AP mixture (tablet)	Placebo tablet, 400mg, tid, 3d	Severity of symptom sum score	3
Melchior et al. 2000 Pilot [79] Sweden	Uncomplicated URTIs	Within 36h	TG: 39, range: 30–48; CG: 42.8, range: 32–52	TG: 35%; CG: 39%	45/46	AP mixture (tablet)	Placebo tablet, 400mg, tid, 3d	Severity of symptom sum score; Cough (frequency/dry/productive); Sore throat improvement score	4–6

NR: not reported, TG: treatment group, CG: control group, SD: standard deviation, Y: year, m: month, d: day, h: hour. AP: *A*. *Paniculata*, URTIs: upper respiratory tract infections, AURTIs: acute upper respiratory tract infections, Qd: once daily, bid: twice daily, tid: three times daily, qid: four times daily, po: oral. PP: per-protocol, ITT: intention-to-treat. CCME: cure and markedly effective rate (not reported as guideline based)

**Table 2:** Trial characteristics: *A*. *Paniculata* versus Usual care (n = 12).

STUDY ID	Diagnosis (syndrome differentiation)	Course of symptoms: mean±SD	Age: Mean±SD (y)	Gender (% of male)	N (analysed/recruited)	Name of the TG product & co-intervention if available	Details of CG	Outcome measures	End point
Chang 2012 [50] China	AURTIs	1.5d, range: 0.5–3d	38.5 (15–65)	44%	64/64	AP mono (granule)	Ribavirin, iv, 10mg/kg in 250ml 5% Glucose solution, qd; penicillin, cefazolin; for 3–7d)	CCME	3–7
Li 2014 [51] China	Acute pharyngitis (Hou Bi)	NR	TG: 30.5±1.7; CG: 29.8±1.8	TG: 68%; CG: 60%	52/52	AP mono (pillule)	Cefixime capsule, 400mg, qd, 7d/session, 2 sessions	CCME	20
						One off treatment: inhalation of small amount Glucocorticoids (dosage N/A), healthy diet, no alcohol or cigarettes			
Thamlikitkul et al 1991 [52] Thailand	Pharyngotonsillitis	NR; "recent fever" (inclusion criteria)	TG1: 29.3±8.1; TG2: 29.4±6.4; CG: 28.2±7.4	TG1: 51%; TG2: 48%; CG: 53%	142/152	AP mono (capsule); TG1: HAP; TG2: LAP	Paracetamol capsule, 325mg, qid, 7d	CCME (sore throat)	3
						Antibiotic, antihistamine or/and decongestant, antitussive			
Hou et al 2009 [53] China	AURTIs:	Within 3d (inclusion criteria)	*TG: 21.87±19.92; CG: 21.33±14.05 (m)	TG: 59%; CG: 61%	397/397	AP mixture (capsule)	Ribavirin; 6d	CCME	NR, probably 6
Lin and Yang 2011 [54] China	Herpes Anginosus	NR; participants all had sudden fever	*Range: 6m–7y	51%	98/98	AP mixture (capsule)	Ribavirin	**CCME	7
						Antipyretic or physically cooling down; antibiotics (If WBC > 10x10(9)/L-); IV fluid infusion (if participants couldn't eat)			
Liu et al 2012 [55] China	AURTIs	NR	TG: 41.56, range: 20–63; CG: 41.87, range: 20–65	TG: 48.33%; CG: 50.82%	121/121	AP mixture (capsule)	Ribavirin granule, 0.3g, tid, 7d	CMCRG-CCME; Time to resolution (cough and sore throat)	7
						Anti-infection, anti-cough, and antipyretic			
Tan and Gao 2010 [56] China	ARTIs (wind heat)	TG: 1.71±0.46; CG: 1.67±0.48	TG: 40.3±11.43; CG: 38.45±12.36	TG: 55%; CG: 56%	124/144	AP mixture (capsule)	Ribavirin, 0.3g, tid, 3d	[FAS data] CCME; Symptom improvement (cough and sore throat); Time to resolution (cough)	3, 7
						Drink plenty of water, saline gargle, bid; Phenol caplets, po, 2 tablets, tid; Fu Fang Gan Cao He Ji (if cough), po, 10ml, tid; Physical cooling down (if >38°C); Benorilate, po, 1g (if >39°C)			
Tan 2011 [57] China	URTIs—group B coxsackieviruses (wind heat)	TG range: 7–14d; CG range: 8–14d	TG median: 27; CG median: 28	TG: 47.83%; CG: 41.3%	92/92	AP mixture (capsule)	Ribavirin tablet; 0.3g, tid, 7d	CMCRG-CCME	7
						Drink plenty of water, rest; physically cooling down (if > 38°C)			
Wang et al 2008 [58] China	ARTIs	NR	TG: 42.38±1.12; CG: 42.56±1.44	TG: 52.22%; CG: 49.44%	324/347	AP mixture (capsule)	Ribavirin granule	**CMCRG-CCME; Time to resolution (overall symptoms)	6
						Dry suspension of cefaclor (if bacterial infection)			
Kulichenko et al., 2003 [59] Russia	Diagnosed Influenza viral infection	NR	Range: 19–63	NR	66/66	AP mixture (tablet) + paracetamol (if >39°C)	Amantadine "according to prescription", regimen not clearly stated but possibly same as in the pilot study listed below	Cough and sore throat (Patient's self-evaluation (scale 0–3); Sore throat (Patient's self-evaluation (scale 0–3); Time to resolution (cough and sore throat)	5
Pilot [59] Russia	Diagnosed Influenza viral infection	NR	Range: 19–63	NR	540/540	AP mixture (tablet) + paracetamol (if >39°C)	Antiviral (Amandine with ascorbic acid as an adjuvant). 1st day: 2*0.05g tablet, tid; 2nd & 3rd day: 2*0.05g tablet, bid; 4th day: 2*0.05g tablet, qd. Paracetamol (if > 38°C), 1*0.05 g tablets, tid, 2–3d	CCME (cough and sore throat); Days of sick leave; Time to resolution (cough and sore throat)	4–5
Li 2010 [60] China	ARTIs (Feng Wen Re Du)	TG: 7d; CG: 8d	*TG: 9±1.5; CG: 8±1.7	TG: 69%; CG: 70%	130/130	AP mixture (tablet)	Aciclovir tablets, po, 0.8g, 5 times a day; Vitamin C, po, 0.2g, tid	**CCME	NR; probably 7
						Ru Yi Huang Jin San (external use, Cu Tiao) and health advice (avoid sun and wind; no spicy or strong flavour food)			
Deng 1999 [61] China	Acute tonsillitis	2h-7d	*TG: 5–62; CG: 5–62	TG: 52.58%; CG: NR	162/162	AP mixture (liquid)	Erythromycin ethylsuccinate; 250–500mg, tid-qid (children: 30–50ml/kg, tid-qid), 7d	CCME; Time to resolution (overall symptoms)	7

*Trials on or involved children;

**Practitioner evaluated

NR: not reported, TG: treatment group, CG: control group, SD: standard deviation, Y: year, m: month, d: day, h: hour. AP: *A*. *Paniculata*, HAP: high dose *A*. *Paniculata*, LAP: low dose *A*. *Paniculata*. URTIs: upper respiratory tract infections, AURTIs: acute upper respiratory tract infections. Qd: once daily, bid: twice daily, tid: three times daily, qid: four times daily, po: oral. FAS: full analysis set, PP: per-protocol, ITT: intention-to-treat. CCME: cure and markedly effective rate (not reported as guideline based). CMCRG-CCME: cure rate and markedly effective rate based on the Chinese medicine clinical research guidelines

**Table 3:** Trial characteristics: *A*. *Paniculata* plus usual care versus Usual care (n = 9).

STUDY ID	Diagnosis (syndrome differentiation)	Course of symptoms: mean±SD	Age: Mean±SD (y)	Gender (% of male)	N (analysed/recruited)	Name of the TG product & co-intervention if available	Details of CG	Outcome measures	End point
Bao 2013 [62] China	Acute pharyngitis	NR	TG: 23.6±1.2; CG: 22.4±1.9	TG: 60%; CG: 57.5%	40/40	AP mono (pillule)+ usual care	Usual care: Corticosteroids combined with antibiotics (Gentamicin and dexamethasone), 1 ml for 15 mins/d, 5d; Cydiodine Buccal tablets, 1.5mg, tid, 5d	CMCRG-CCME	5
Sun and Zhao 2014 [63] China	Bronchiectasis (Fei Yong)	NR; “Acute exacerbation”	TG: median: 49.2, range: 21–80; CG: median: 50.1, range: 22–78	TG: 46%; CG: 51%	78/78	AP mono (capsule) + usual care	Usual care: Cefixime, po, 150mg, bid; Levofloxacin, po, 0.2g, bid; Dextromethorphan hydrobromide and guaifenesin syrup, po, 20ml, tid; all for 14d	Severity of cough (VAS, 0–10)	11
Guo 2013 [64] China	ARTIs (External wind heat)	Within 3d	*TG: 5.25±1.42; CG: 5.43±1.39	TG: 61%; CG: 58%	416/416	AP mixture (capsule) + Ribavirin	Ribavirin	**CCME	NR, probably 7
Li et al 2007 [65] China	Pneumonia	10.5 (range: 7–14)	*Range: 1m–5y	TG: 58.33%; CG: 60%	540/540	AP mixture (capsule) + usual care	Usual care: Antibiotics and antivirals; Aminophylline; Vitamin K; Sedation, diuretic, cardiac, oxygen (if heart failure); Dehydrating agent and brain cell activator (if toxic encephalopathy)	**CCME	NR, probably 7
Meng 2012 [66] China	Acute tracheitis and bronchitis	Within 5d (as inclusion criteria)	NR	NR	282/282	AP mixture (capsule) + usual care	Usual care: Drink more water, rest, gargle bid; If there were symptoms of URTIs such as nasal congestion, runny nose, or sneezing, Paracetamol Triprolidine Hydrochloride and Pseudoephedrine Hydrochloride tablets were given, po, 2 tablets, tid; If cough with no or little sputum, Pentoxyverine Citrate Tablets was given, 250mg, po, tid; If cough with sputum, Bisolvon Tablets was given, po, 160mg, tid; If fever, physical cooling; If there was clear evidence of bacterial infection, antibiotics such as macrolides, penicillins, cephalosporins, or quinolones were used	**CCME; Severity of cough	7
Tang et al 2009 [67] China	Bronchitis	Range: 1–2d	*7.5m, range: 3–12m	56%	260/260	AP mixture (capsule) + usual care	Usual care: Anti-infection, sedation, ultrasonic atomization, sputum suction, shoot back	**CCME; Time to resolution (cough)	7
Wu 2013 [68] China	Acute bronchitis	5.4 ±3.6, range: 1–13	9–73, 34.2 ± 11.2	53%	362/362	AP mixture (capsule) + usual care	Usual care: Paracetamol Triprolidine Hydrochloride and Pseudoephedrine Hydrochloride tablets, po, 2 tablets, tid; Pentoxyverine Citrate tablets, po, 250mg, tid; Bromhexine, po, 160mg, tid	**CCME	7
Shakhova et al 2003 [69] Russia	URTIs	Within 24h	*NR; children	NR	93/93	AP mixture (tablet) + usual care	Usual care: drink plenty of warm water; milk and vegetable diet with food containing vitamins; deep throat rinse with Alkaline and mouth washing; 1–2% solution of protargola (silver proteinate); paracetmal	Severity of symptom sum score	3–5 and 7–9
Spasov et al., 2004 [70] Russia	URTIs	Within 24h (inclusion criteria)	*TG: 7.17±0.32; CG1: 6.78±0.34; CG2: 6.47±0.29	TG: 49%; CG1: 49%; CG2: 56%	133/133	AP mixture (tablet) + usual care	CG1: Immual (Echinacea purperea) drop + usual care; CG2: Usual care (lavish warm drinks, throat gargles, antiseptic nose drops, and paracetamol, 500mg, tid (if fever or severe headache)	Severity of symptom sum score (patient and practitioner evaluated), reduce in medications	5

*Trials on or involved children;

**Practitioner evaluated

NR: not reported, TG: treatment group, CG: control group, SD: standard deviation, Y: year, m: month, d: day, h: hour. AP: *A*. *Paniculata*, HAP: high dose *A*. *Paniculata*, LAP: low dose *A*. *Paniculata*. URTIs: upper respiratory tract infections, AURTIs: acute upper respiratory tract infections. Qd: once daily, bid: twice daily, tid: three times daily, qid: four times daily, po: oral. FAS: full analysis set, PP: per-protocol, ITT: intention-to-treat. CCME: cure and markedly effective rate (not reported as guideline based). CMCRG-CCME: cure rate and markedly effective rate based on the Chinese medicine clinical research guidelines

**Table 4:** Trial characteristics: *A*. *Paniculata* versus Herbal active intervention (n = 5).

STUDY ID	Diagnosis (syndrome differentiation)	Course of symptoms: mean±SD	Age: Mean±SD (y)	Gender (% of male)	N (analysed/recruited)	Name of the TG product & co-intervention if available)	Details of CG	Outcome measures	End point
Ding et al 2010 [71] China	Acute bronchitis (wind heat)	TG: 2.76±1.03d; CG: 2.80±1.18d	TG: 37.68±13.25; CG: 34.96±13.32	TG: 53%; CG: 38%	136/137	AP mixture (capsule) + CG placebo	Qing Gan Chuan Xin Lian tablet (Chuan Xin Lian + Mai Ma Teng), 0.25g, tid + TG placebo	**CMCRG-CCME	0, 2, 3, 4, 8
	ARTIs (wind heat)	TG: 18.91±9.85h; CG: 18.63±12.24h	TG: 35.97±13.12; CG: 33.27±12.57	TG: 43%; CG: 40%	138/140	Same as above			
Xi 2006 [72] China	Cold (Shu Shi)	Within 3d (inclusion criteria)	TG: 36±2.26; CG: 35±2.12	TG: 56%; CG1: 56%; CG2: 50%	250/250	AP mixture (tablet)	CG1: Huo Xiang Zheng Qi pill, 6–8 pills, tid, 3d; CG2: Su Xiao Shang Feng capsule, 2 capsules, tid, 3d	CMCRG-CCME	3
Yang and Liu 2012 [73] China	URTIs (wind heat)	Within 48h (within 24h: n = 160)	TG: 35.47; CG: 34.56 (SD NR)	TG: 43%; CG: NR	233/239	AP mixture (tablet)	Fu Fang Yu Xing Cao tablet; 4 tablets, tid, 3d	CMCRG-CCME	3
Zhang et al 1994 [74] China	Acute tonsillitis (criteria given)	Within 3d	*TG: <10: n = 47, >10: n = 54; CG: <10: n = 21; >10: n = 32	TG: 60%, CG: 53%	154/154	AP mixture (liquid)	Yin Huang liquid: Jin Yin Hua extract 12g + Huang Qin extract 24g, 10ml, tid, 7 days (children half dose)	CCME	7
Zhao et al., 2012 [75] China	Common cold (wind heat)	Within 48h (inclusion criteria)	TG: 30.7; CG: 31.1 (SD NR)	TG: 50%; CG: 50%	300/300	AP mixture (granule)	Gan Mao Ling granule; one pack, tid, 5d	CMCRG-CCME; Severity of symptom score (cough and sore throat)	5

*Trials on or involved children;

**Practitioner evaluated

NR: not reported, TG: treatment group, CG: control group, SD: standard deviation, Y: year, m: month, d: day, h: hour. AP: *A*. *Paniculata*, HAP: high dose *A*. *Paniculata*, LAP: low dose *A*. *Paniculata*. URTIs: upper respiratory tract infections, AURTIs: acute upper respiratory tract infections. Qd: once daily, bid: twice daily, tid: three times daily, qid: four times daily, po: oral. FAS: full analysis set, PP: per-protocol, ITT: intention-to-treat. CCME: cure and markedly effective rate (not reported as guideline based). CMCRG-CCME: cure rate and markedly effective rate based on the Chinese medicine clinical research guidelines

**Table 5:** Trial characteristics: *A*. *Paniculata* (pillule) versus *A*. *Paniculata* (tablet) (n = 3).

STUDY ID	Diagnosis (syndrome differentiation)	Course of symptoms: mean±SD	Age: Mean±SD (y)	Gender (% of male)	N (analysed/recruited)	Name of the TG product & co-intervention if available	Details of CG	Outcome measures	End point
Chang et al 2008 (phase 1) [80] China	ARTIs (External wind heat)	TG: 22.44±12.22h; CG: 20.7±8.46h	TG: 36.31±11.63; CG: 37.55±12.69	TG: 57%; CG: 62%	200/202	AP mono (pillule)	Chuan Xin Lian tablet, 0.15g; tid; 3d	[FAS data] CMCRG-CCME	0, 2, 4
(phase 2) [80] China	ARTIs	NR	TG: 37.18±13.64; CG: 36.09±14.43	TG: 48.55%; CG: 46.32%	271/274/276	AP mono (pillule)	Chuan Xin Lian tablet, 0.15g; tid; 3d	[FAS data] CMCRG-CCME	0, 2, 4
Su 2014 [81] China	Acute pharyngitis	NR	26.5 (range: 20–40)	53%	60/60	AP mono (pillule)	Chuan Xin Lian tablet; 1g, tid, 5d	CMCRG-CCME	5
						Inhalation of Gentamicin 80,000 ∪, dexamethasone 5mg; 15 mins, bid, 5d			
Xia 2014 [82] China	Acute pharyngitis	NR	TG: 35.6, range: 16–68; CG: 36.4, range: 17–63	TG: 55%, CG: 52%	125/125	AP mono (pillule)	Chuan Xin Lian tablet, 0.3g, tid, 3–7d	CMCRG-CCME	3–7

NR: not reported, TG: treatment group, CG: control group, SD: standard deviation, Y: year, m: month, d: day, h: hour. AP: *A*. *Paniculata*, HAP: high dose *A*. *Paniculata*, LAP: low dose *A*. *Paniculata*. URTIs: upper respiratory tract infections, AURTIs: acute upper respiratory tract infections. Qd: once daily, bid: twice daily, tid: three times daily, qid: four times daily, po: oral. FAS: full analysis set, PP: per-protocol, ITT: intention-to-treat. CCME: cure and markedly effective rate (not reported as guideline based)

Twenty-two trials [50–55, 57, 59, 61, 62, 69, 70, 72–79, 81, 82] were on upper ARTIs; while six trials on lower ARTIs were published in China [63, 65–68, 71]; and six did not specify upper or lower [56, 58, 60, 64, 71, 80]. Eleven trials [55, 57, 58, 62, 71–73, 75, 80–82] reported the use of guideline based diagnosis, according to the Chinese medicine clinical research guidelines (CMCRG) [中药新药临床研究指导原则] [83]; the international classification of primary care (ICPC) classification [84]; the criteria of diagnosis and therapeutic effect of diseases and syndromes in traditional Chinese medicine [中医病证诊断疗效标准] [85]; and the common clinical diseases and diagnosis criteria [常见疾病诊断依据与疗效判断标准] [86].

Nearly one third of the trials did not include patients with a co-morbidity or did not report existence of a co-morbidity, but they excluded patients who had other primary diseases [50, 52, 53, 56, 59, 71, 77–80], e.g. cardiovascular conditions, liver, kidney or hematopoietic system impairment, mental health conditions, or rheumatoid arthritis. Two trials excluded patients who had asthma [52, 77]; two excluded those who had any other infections [76, 78]. Only three trials included patients with co-morbidities: heart failure [65, 67], diarrhoea [58], and toxic encephalopathy [65]; and one trial recruited children with frequent cold, bronchitis, sinusitis and pneumonia [69].

### Interventions

Experimental interventions included *A*. *Paniculata* as a monotherapy and as an herbal mixture in combination with other herbs. Table 6 presents the characteristics of *A*. *Paniculata* reported in the included trials. Out of the 33 trials, seven did not report the type of product used [50, 54, 61, 73, 75, 81, 82] whilst one used dried leaves of *A*. *Paniculata* [52]. The remaining 25 trials [51, 53, 55–72, 76–80] reported using *A*. *Paniculata* extract and among these five reported the use of an extract by the name of SHA-10 [59, 70, 76, 78, 79].

**Table 6:** Characteristics of *A*. *Paniculata* used in the included trials.

Name	Ingredient	Form	Manufacturer	ID	Active content and dose strength(s)	Treated condition (syndrome differentiation if available)	Regimen
**Ke Gan Shuang Qing**	Huang Qin Gan, Chuan Xin Lian Nei Zhi	Capsule	Chengdu Kanghong Pharmaceuticals Group Co., Ltd	[71]*	Baicalin: Andrographolide ratio 4:1 (100mg and 25mg)	Acute bronchitis (Wind heat) & ARTIs	125mg, 3 capsules, tid, 4d
				[56]*	Baicalin: Andrographolide ratio 4:1 (150mg:37.5mg)	AURTIs	375mg, tid, 3d
				[57]*		URTIs—group B coxsackieviruses (Wind heat)	2 capsules, tid, 7d
				[55]	Baicalin and Andrographolide 4:1	AURTIs	2 capsules, tid, 7d
				[68]*	NR	Acute bronchitis	2 capsule, tid, 7d
				[66]*		Acute tracheitis and bronchitis	2 capsule, tid, 7d
			NR	[67]	Baicalin: Andrographolide ratio 4:1 (150mg:37.5mg)	Bronchiolitis	2 capsules, tid, 7d
				[64]	Baicalin and Andrographolide 4:1	Acute RTIs (External wind heat)	1 capsule, tid, 7d
				[58]	NR	ARTIs	2 capsules, tid, 6d
				[53]		AURTIs	1 capsule, tid, 6d
				[54]		Herpes Anginosus	1 capsule, tid, 5–7d
				[65]		Pneumonia	Tid, “till discharge"
		Granule	NAP	[50]	10g Chuan Xin Lian + 10g Huang Qin	ARUTIs	Qid, 3–7d
**Fu Fang Shuang Hua**	Chuan Xin Lian, Yin Hua, Lian Qiao, Ban Lan Gen	Tablet	Shanxi Kanghui Pharmaceutical Co., Ltd	[73]*	NR	URTIs (Wind heat)	4 tablets, qid, 3d
				[72]*	NR	Cold (Shu Shi)	4 tablets, tid, 3d
				[60]	NR	ARTIs (Feng Wen Re Du)	4 tablets, tid, course of treatment NR
		Liquid	Beijing Haierfu Pharmaceutical Co., Ltd	[61]	NR	Acute tonsillitis	5–7 yrs: 10ml, qid; children above 7 yrs: 20ml, tid; adult: 20ml, qid; 7d
			NR	[74]	NR	Acute tonsillitis	For children (<3yrs: 10ml, tid; 3–7yrs: 10ml, qid; >7yrs: 20ml, tid); For adult (20ml, qid); 7d
**Kan Jang**	Elethrococcus senticosus, *A*. *paniculata*	Tablet	The Swedish Herbal Institute, Goteborg, Sweden	[69]	Elethrococcus senticosus and AP	URTIs	2 tablets, tid, 5–7d
				[79]*	AP extract (EX20101) 85mg, SHA containing 5.25mg Andrographolide and deoxyandrographolide per tabet; Acanthopanax senticosus EX20095 9.7mg containing total Eleuthroside B and Eleuthroside E 2%	Uncomplicated URTIs	Main: 4 tablets (400mg), tid, 3d; pilot: 4 tablets (400mg), tid, 4–6d
				[59]*	88.8mg AP; Eleuthrococcus senticosus 10.0mg	Influenza viral infection	300mg, tid, 5d
				[70]*	85mg of AP containing 5.25mg andrographolide and deoxyandrographolide and extract of Eleuthrococcus senticosus EX20095, 9.7mg	URTI	200mg, tid, 5d
**Jun Du Qing**	Ban Lan Gen, Xuan Shen, Qian Cao, Dan Shen, Jin Yin Hua	Granule	Sun Yat-sen university affiliated hospital	[75]*	NR	Common cold (Wind heat)	2 packs, tid, 5d
**Chuan Xin Lian Nei Zhi**	*A*. *Paniculata* monotherapy	Pillule	Tianjin Tasly Pharmaceutical Co., Ltd	[80]*	NR	ARTIs (External wind heat)	0.15g, tid, 3d
				[51]*	NR	Acute pharyngitis (Hou Bi)	0.15g, tid, 7d
				[62]*	NR	Acute pharyngitis	0.15g, tid, 5d
		Capsule	Jiuhui Pharmaceutical Co., Ltd	[63]*	75mg Andrographolide/capsule	Bronchiectasis (Fei Yong)	0.33g, tid, 14d
**Chuan Xin Lian**		Pillule	Sichuan Herun Pharmacy Co., Ltd	[82]*	NR	Acute pharyngitis	630mg (42mg/pillule X15 pillule), tid, 3–7d
				[81]	NR	Acute pharyngitis	630mg, tid, 5d
**Kang Jang**		Tablet	The Swedish Herbal Institute	[77]*	Each tablet contained 85mg of AP	Common cold	400mg, tid, 5d
				[76]*	100mg each of AP herb dried extract; Standardised to a minimum of 5mg of total andrographolide and deoxyandrographolide	Common cold	4 tablets, tid, 5d (1200 mg/day of A *paniculata* dried extract)
**KalmCold™**		Capsule	M/s Natural Remedies Pvt. Ltd. Bangalore, India	[78]*	200 mg of KalmCold dissolved in 100 ml of Methanol	Uncomplicated URTI	one capsule (100 mg active component), bid after breakfast and dinner, for 5d
**LAP/HAP**		Capsule	The Department of Medical Science, Ministry of Public health	[52]	HAP: 500 mg AP per capsule (casule of 500 mg); LAP: 250 mg AP per capsule (capsule of 250 mg)	Pharyngotonsillitis	HAP: 3 capsules 4 times a day during 7d: 6g of Andrographis a day, LAP: 3 capsules 4 times a day during 7d: 3g of Andrographis a day

*Products with authentication information provided

NAP: not a product. NR: not reported, TG: treatment group, CG: control group, d: day, yrs: years. AP: *A*. *Paniculata*, HAP: high dose *A*. *Paniculata*, LAP: low dose *A*. *Paniculata*. URTIs: upper respiratory tract infections, AURTIs: acute upper respiratory tract infections. Qd: once daily, bid: twice daily, tid: three times daily, qid: four times daily, po: oral

Included trials seldom reported manufacturing or quality control details. Three reported method of measuring andrographolide proportion using HPLC technique [76–78]; and only one reported that the product was produced, analysed and bottled according to good manufacturing practice (GMP) standard [59]. Three trials reported added materials [57, 76, 78] but only one [78] provided clear description (200 mg of micro crystalline cellulose). Extract solvents used included methanol [78], polyethylene glycol [80], and two used methanol for HPLC extraction [76, 77]. Only one trial provided extract solvent concentration details [78].

Comparison interventions included usual care, placebo control, and active herbal interventions. All the 21 trials involving usual care [50–70] included some form of active intervention such as corticosteroids [51, 62], antibiotics or antivirals [50, 53, 55, 57–59, 61–67], cough suppressant [55, 56, 63, 65, 66, 68], or antipyretics [52, 54–56, 60, 66, 68–70].

### Outcome measurements

The most commonly reported primary outcome measure was global assessment on overall symptoms improvement (Tables 1–5). Although not clearly reported in every trial, it is assumed this outcome was measured by the practitioner. Apart from one study [63], all Chinese-language trials reported four-category scores in symptoms of ARTIs, among which 11 [55, 57, 58, 62, 71–73, 75, 80–82] reported data based on the CMCRG [中药新药临床研究指导原则]. The CMCRG is a four-category scoring system to evaluate overall treatment effects based on: 1). Cured: a). no temperature in 3 days, b). no symptom or sign of RTIs, c). accumulated score decreases ≥95%; 2) Markedly effective: a). no temperature in 3 days, b). most symptoms and signs of RTIs disappear, c) accumulated score decrease between 70% to 95%; 3). Effective: body temperature decreased in 3 days, b). most of key symptoms and signs of RTIs disappear, c). accumulated score decrease between 30% to 70%; 4). Ineffective or worsening: a). no decrease or increased body temperature, b). no improvement in key symptoms and signs of RTIs or even getting severe, c). accumulated score decreases less than 30%. Accumulated score was calculated as: (baseline score—endpoint score)/baseline score X100%. Scores were given based on: 1). Symptoms of ARTIs, e.g. symptoms: fever, sore throat, cough, nasal congestion, runny nose, headache, sweating, sneezing, thirst, 2). Signs of ARTIs, e.g. aversion to wind, and changes in tongue appearance and pulse; and 3). Laboratory checks, e.g. chest radiography, circulation, faeces, blood, urine, liver and kidney function, electrocardiogram (ECG). In this review, the combined cure and markedly effective (CCME) rate was considered as improved by the review authors. Symptom score on severity of cough [59, 63, 66, 75, 76], sore throat [59, 75], and overall symptoms (commonly a list of 8–12 ARTI symptoms) [69, 70] were reported in seven trials.

Secondary outcome measures reported in the included trials were: time to resolution of cough [55, 56, 59, 67], of sore throat [55, 56, 59], and of overall symptoms [58, 61]; only one trial reported reduction in reported antibiotic usage [70].

A few trials used a repeated measures approach [50, 56, 69, 71, 80]. Apart from one trial on acute pharyngitis which followed-up at 20 days [51], the most common end point follow-up that was reported ranged from 3 to 7 days and the outcome data for the end points closest to an average of 5 days were extracted and assessed (Tables 1–5).

### Risk of bias of included trials

**Fig 2:**
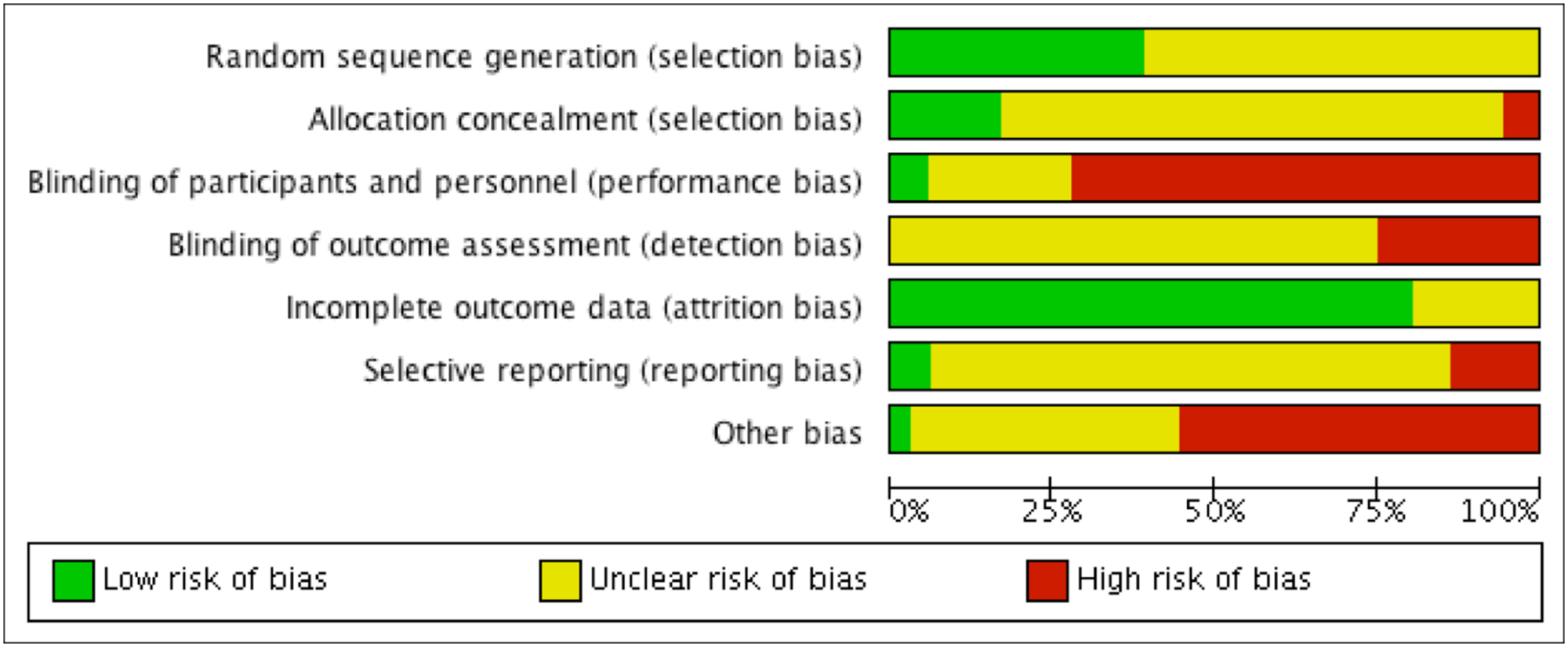
Risk of bias graph: Review authors' judgements about each risk of bias item presented as percentages across all included trials.

Apart from four trials [52, 76, 78] (and pilot of [79]), all other trials were judged at high risk of bias on at least one domain (Fig 2). Each risk of bias item for each included trial are provided in supplement information; see S1 Fig.

All included trials were described as ‘randomised’, but 20 did not report the method of random sequence generation [50, 52–55, 57, 58, 60–62, 65, 67, 69, 72–74, 77, 79, 81, 82]. Among those that did, seven used random number table [51, 63, 64, 68, 71, 75, 80] and six used computer-generated random series [56, 59, 66, 70, 76, 78]. Only four trials provided information on allocation concealment, among these two were organised by independent third party clinical management personnel [78, 80], and two used sealed identical jars [76, 79].

Most trials (24 of 33) had a high risk of bias in blinding of the participants and personnel as they assessed two interventions that were different in dosage, or form of preparation, or two types of interventions, or compared *A*. *Paniculata* plus usual care versus usual care, without any blinding information given. Two trials comparing *A*. *Paniculata* with placebo control had low risk of bias as both patients and evaluator [76] or investigator and pharmacist [78] were blinded to group assignment and could not distinguish between the two interventions. The remaining trials [52, 55, 59, 71, 74, 77, 79] provided no information regarding similarities between interventions, or provided no information to confirm whether or not blinding of personnel was conducted.

Most included trials failed to provide enough information to determine whether blinding of outcome assessment was achieved. Nine trials were judged to be at high risk of bias as they assessed subjective outcome measures and the patients or practitioners knew that which intervention they had been assigned to (i.e. *A*. *Paniculata* plus usual care versus usual care) [62–70].

Twenty-six included trials reported no attrition. Among the 7 trials that had dropouts, three trials reported 3–8% dropout and conducted ITT by assuming no effect for dropouts. No per protocol analysis was performed for those three trials [56, 58, 73]. Two trials reported dropouts (1% [78] and 6% [52]) without ITT analysis. Another trial reported 25% dropout and provided both ITT and per protocol analysis findings [76]. The author suggested that the dropout rate in two groups were equal and that the potential reason for large dropout may have been related to three weeks’ winter holiday. One trial did not clarify how missing data was dealt with [70].

One trial [79] published a protocol containing information on outcome measures and follow-up points that were consistent with the main trial report. All remaining trials did not have a protocol available. Four trials [65, 71, 75, 82] reported selected findings that were not fully consistent with the outcome measures set in the methods.

Only one trial had no obvious risk of other bias [80] and this was the only trial that stated that there was no conflict of interests. None of the other included trials stated whether or not a conflict of interest existed and three trials included one or more author who worked for the pharmaceutical company of the product being evaluated as an intervention [59, 71, 77]. The most common reasons for high risk of other bias were: 1). In 12 trials, diagnostic criteria were not applied at recruitment and there were no inclusion or exclusion criteria specified [53, 54, 58, 60–62, 65, 67, 68, 74, 81, 82]; 2). Four trials provided either no condition-related baseline data [63, 75, 81, 82], or no sociodemographic characteristic baseline [59, 79], or neither [69]; and 3). Two trials reported discrepancies between permitted co-intervention(s) for the intervention and control groups: in one trial, paracetamol was given if body temperature > 39 in the treatment group but 38–38.5 in the control group [59]; the other trial allowed no additional treatment for the intervention group only [61]. One third of the trials reported informed consent [55, 56, 59, 64, 66, 69–71, 78–80].

**Fig 3:**
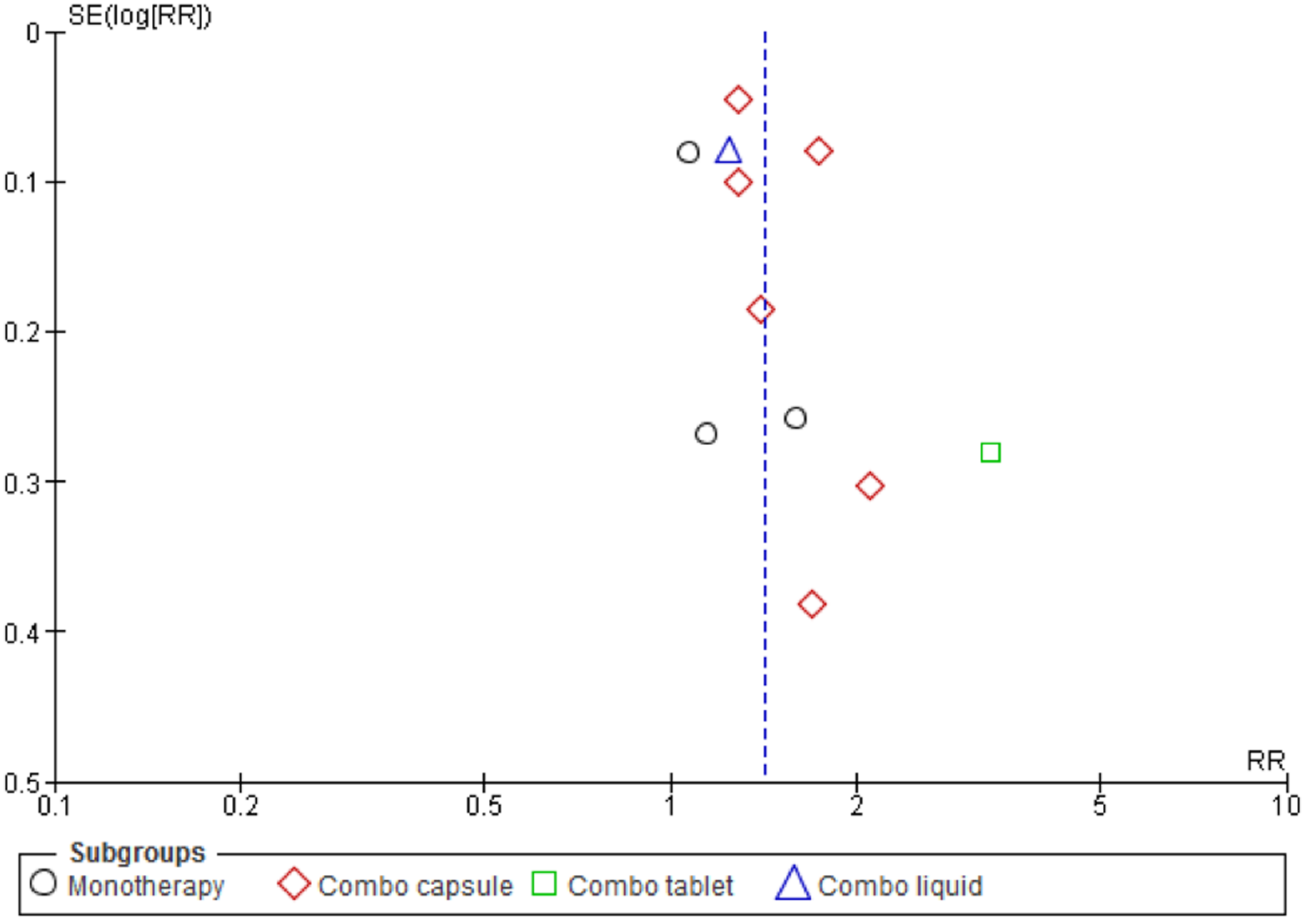
Funnel plot of comparison: 1 *A*. *Paniculata* vs. Conventional active intervention, outcome: 1.1 Chinese guideline assessment of symptom improvement.

Funnel plot for one comparison was performed to investigate potential publication bias (Fig 3). There was no evidence (p = 0.870) of small-study effects.

### Effect estimates

The included trials featured five comparison groups: *A*. *Paniculata* versus placebo (4 trials); *A*. *Paniculata* versus usual care (12 trials); *A*. *Paniculata* plus usual care versus usual care alone (9 trials); *A*. *Paniculata* versus other active herbal interventions (5 trials); and *A*. *Paniculata* pillule versus *A*. *Paniculata* tablet (3 trials).

Subgroup analyses were performed for two of the planned subgroups: monotherapy or herbal mixture and different forms of preparation of *A*. *Paniculata*. These were conducted for primary outcome measures in *A*. *Paniculata* versus usual care and *A*. *Paniculata* plus usual care versus usual care. Subgroup analysis in other comparison groups and subgroup analysis on upper or lower ARTIs, and adults versus children were not performed due to insufficient data.

#### *A*. *Paniculata* vs placebo (n = 4)

**Fig 4:**
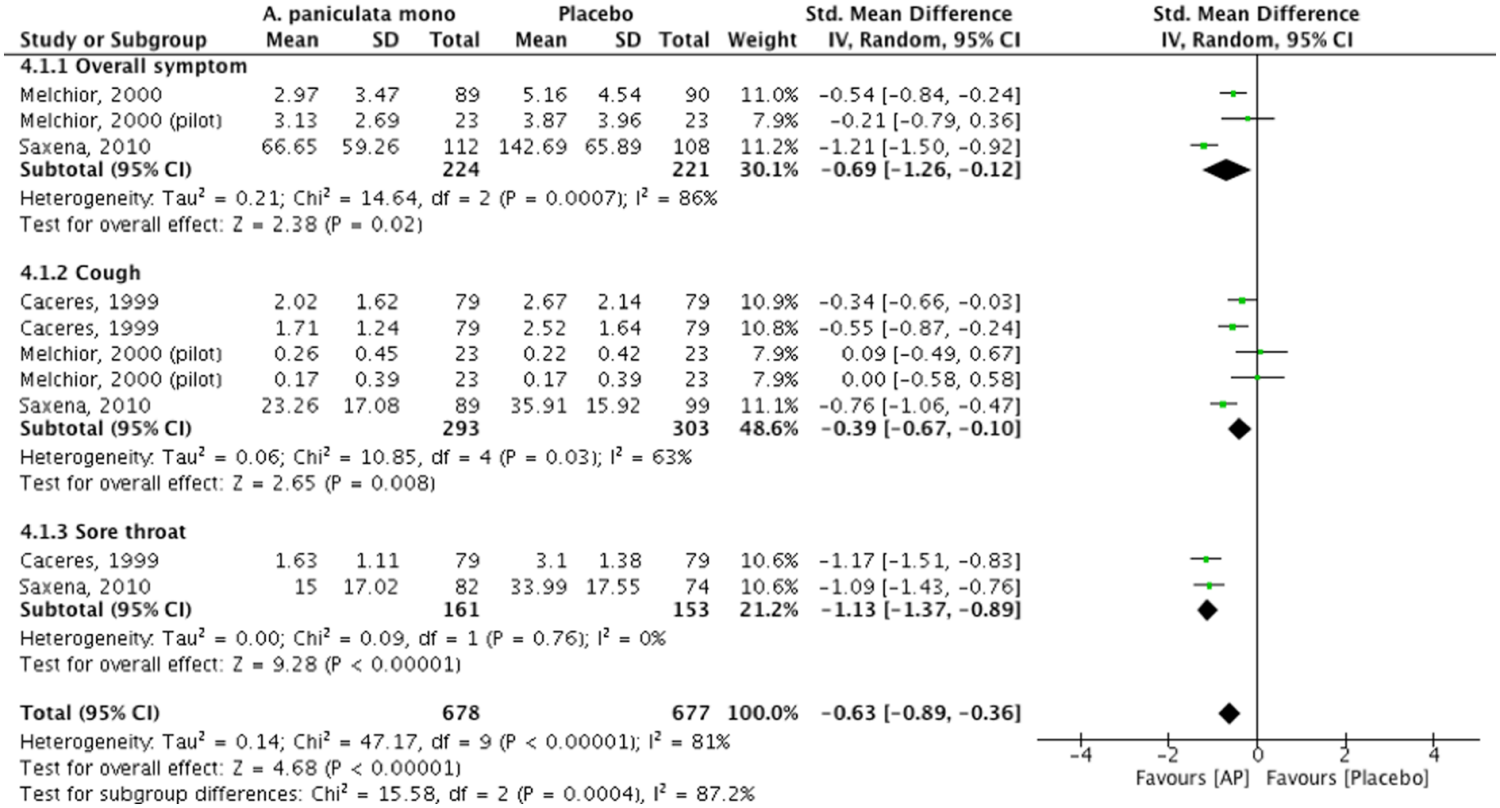
*A*. *Paniculata* versus placebo as measured by symptom improvement score.

Evidence from four trials (three had low or medium RoB [76, 78, 79] showed a statistically significant effect in favour of *A*. *Paniculata* compared to placebo in overall symptom improvement (n = 445, SMD: -0.69, 95%CI [-1.26, -0.12], I^2^ = 86%), cough (n = 596, SMD: -0.39, 95%CI [-0.67, -0.10], I^2^ = 63%), and sore throat (n = 314, SMD: -1.13, 95% CI [-1.37, -0.89], I^2^ = 0%) (Fig 4) [76–79]. One trial showed a statistically significant effect in favour of *A*. *Paniculata* as a single herb in tablet compared to placebo as measured by patient reported rate of improvement in overall symptoms (n = 50, RR: 2.80, 95%CI [1.19, 6.30]) [77]. No data was available under this comparison for time to symptom resolution or antibiotic medication usage.

#### *A*. *Paniculata* vs usual care (n = 12)

**Fig 5:**
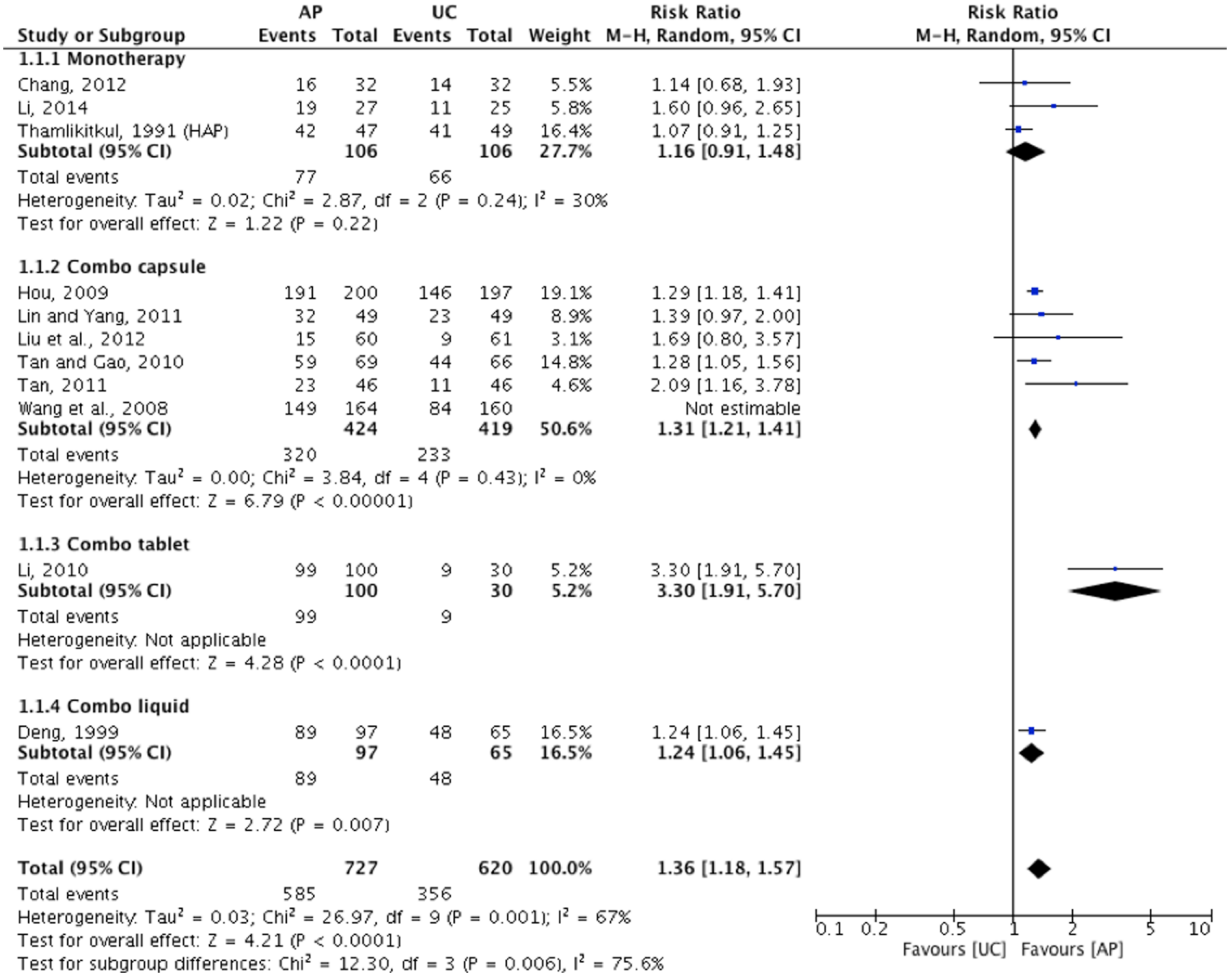
*A*. *Paniculata* versus usual care as measured by global assessment of overall symptoms improvement CCME.

Evidence from ten trials showed a statistically significant effect in favour of *A*. *Paniculata* compared to usual care as measured in overall symptoms improvement CCME rate (n = 1347, RR: 1.36, 95%CI: [1.18, 1.57], I^2^ = 67%) (Fig 5). Heterogeneity for the herbal mixture in capsule subgroup was low when the Wang 2008 trial was removed (p = 0.43, I^2^ = 0%). This may be due to: 1). not reporting inclusion/exclusion criteria for recruiting participants and the duration of illness were not clear, therefore there was potentially high population heterogeneity; and 2) lack of authentication. Apart from one subgroup (*A*. *Paniculata* as a single herb) failing to show a statistically significant effect [50, 51], *A*. *Paniculata* as herbal mixture in capsule [53–58] and as herbal mixture in tablet [60] and liquid [61] showed statistically significant effects compared to usual care.

**Fig 6:**
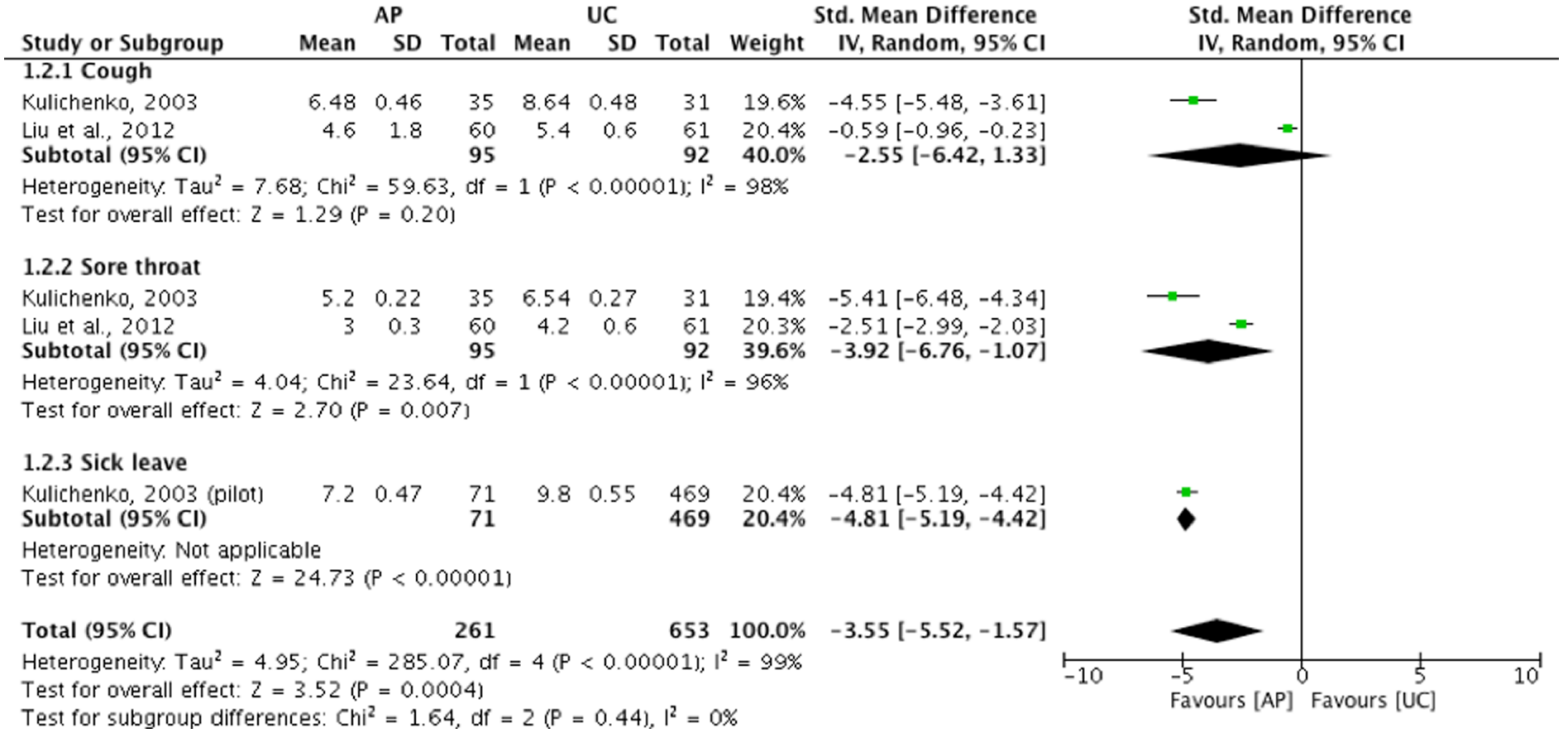
*A*. *Paniculata* versus usual care as measured by time to symptom resolution (Unit: Day).

When compared with usual care, *A*. *Paniculata* showed a statistically significant reduction in the duration of sore throat: (n = 187, SMD: -3.92 [-6.76, -1.07], I^2^ = 96%) and sick leave: (n = 540, SMD: -4.81 [-5.19, -4.42]), but not in cough: (n = 187, SMD: -2.55 [-6.42, 1.33], I^2^ = 98%) (Fig 6) [55, 59]. No data were available on medication usage for this comparison group.

#### *A*. *Paniculata* plus usual care vs usual care (n = 9)

**Fig 7:**
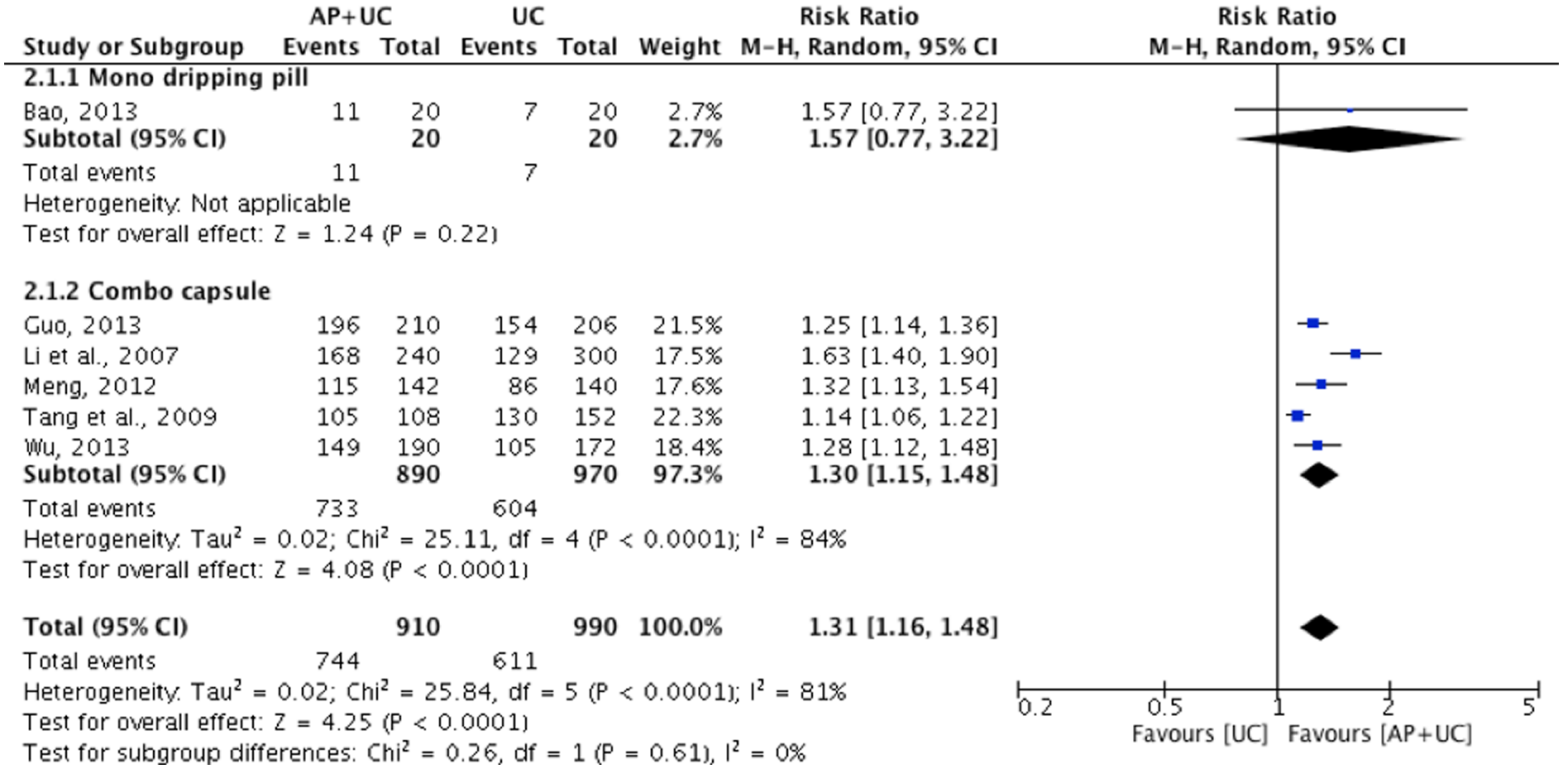
*A*. *Paniculata* plus usual care versus usual care as measured by global assessment of overall symptoms improvement CCME.

Evidence from six trials [62, 64–68] showed a statistically significant effect in favour of *A*. *Paniculata* plus usual care compared to usual care alone as measured by assessment of symptom improvement CCME (n = 1900, RR: 1.31, 95%CI: [1.16, 1.48], I^2^ = 81%) (Fig 7).

**Fig 8:**
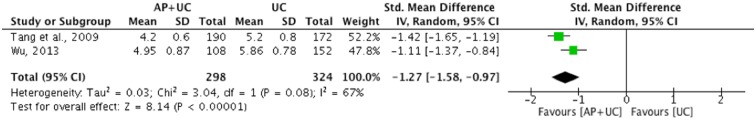
*A*. *Paniculata* plus standard care versus standard care as measured by time to symptom resolution (unit: days).

Evidence from two trials [67, 68] showed that *A*. *Paniculata* plus usual care shortened the duration of symptoms by approximately 1 day compared to usual care alone: (n = 622, SMD: -1.27, [-1.58, -0.97], I^2^ = 67% (Fig 8).

Outcomes of three trials in this comparison group were not pooled and were presented narratively: Sun and Zhao also showed significant improvement in overall symptom as measured by 0–10 VAS (n = 78, MD: -0.80, 95%CI: [-1.40, -0.20]) [63]; Evidence from two trials showed statistically significant improvements in symptoms [69, 70] and Spasov et al. (2004) suggested reductions in paracetamol intake (55 (mean 1.03) over 95 (mean 2.44), p≤0.0001) and codeineintake (23 (mean 0.43) over 43 (mean: 1.10), p≤0.05) when compared *A*. *Paniculata* plus usual care over usual care alone [70].

#### *A*. *Paniculata* vs other herbal interventions (n = 5)

**Fig 9:**
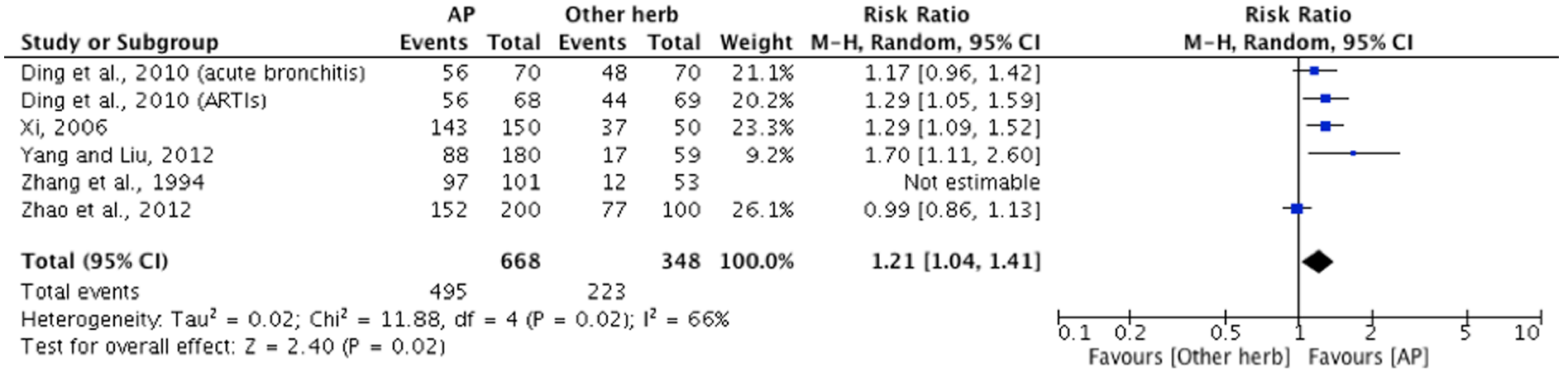
*A*. *Paniculata* versus other herbal interventions as measured by global assessment of overall symptoms improvement.

Evidence from five trials showed a statistically significant effect in favour of *A*. *Paniculata* compared to other herbal interventions as measured by improvement rate in overall symptoms (n = 827, RR: 1.44, 95%CI: [1.10, 1.89], I^2^ = 89%). Upon removing Zhang 1994 from the analysis, heterogeneity was reduced (I^2^ = 66%), while did not greatly change the summary estimates. Possible reasons for this may be that this trial targeted children and that the product evaluated was not authenticated (Fig 9). No data were available for time to resolution or antibiotic medication usage for this comparison group.

#### *A*. *Paniculata* in pillule vs in tablet (n = 3)

**Fig 10:**
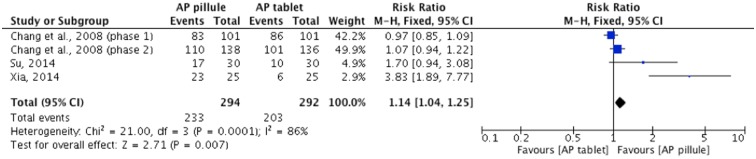
*A*. *Panicualta* pillule versus *A*. *Paniculata* tablet as measured by global assessment of overall symptoms improvement CCME.

Evidence from three trials [80–82] showed a statistically significant effect in *A*. *Paniculata* in pillule compared to *A*. *Paniculata* in tablet as measured by improvement rate in overall symptoms CCME (n = 586, RR: 1.14, 95%CI: [1.04, 1.25], I^2^ = 86%) (Fig 10). No data was available under this comparison for time to symptom resolution or antibiotic medication usage.

### Sensitivity analysis

Sensitivity analyses were conducted by restricting inclusion in the meta-analysis to trials with low risk of bias in both sequence generation and allocation concealment domains [50, 76, 78]. The effect of *A*. *Paniculata* over placebo was enhanced in overall symptoms (n = 219, SMD: -1.21 [-1.50, -0.92]) and in cough (n = 504, SMD: -0.56 [-0.80, -0.31], I^2^ = 46%); while the effect for overall symptoms of using *A*. *Paniculata* in pollule over *A*. *Paniculata* tablet remained similar. Removal of trials that did not provide authentication or standardisation information [50–55, 57, 60, 61, 63–65, 69, 71, 79, 81] did not greatly change the summary estimates. Data from two trials [58, 74] were removed from the meta-analysis with reasons given above.

### Adverse events

All but10 trials [53, 55, 57, 59, 60, 63, 64, 67, 69, 75] reported AE or safety. Among those that reported AEs, none reported any acute toxicity and 11 reported no AE in either intervention or control group [50, 54, 55, 62, 67, 68, 72, 73, 76, 81, 82]. For each of the following AEs associated with the *A*. *Paniculata* group, one case was reported for each trial: constipation [66, 71], nausea [80], vomiting [64], diarrhoea [80], unpleasant sensations in the chest [79], and intensified headache [79] (supplement information; see S2 Table). Four trials did not provide sufficient information to fit into the table are narratively described: Zhang et al. reported some participants had minor AE (vomiting) but did not specify which group or how many participants [74]; Thamlikitkul reported 11 patients in the TG and 9 in CG experienced nausea, vomiting, abdominal discomfort, dizziness, drowsiness and malaise [52]; and Saxena et al reported 1 vomiting, 1 epistaxis, 1 urticarial, 3 diarrhoea (+ nausea or lethargy) [78], and Melchior et al reported 2 cases of urticarial [77], without specifying which group. Saxena et al (2010) stated that the adverse effect between groups were found to be the same (p>0.05) [78].

## Discussion

### Summary of evidence

Thirty-three trials involving 7175 patients with ARTIs were included in this review with no language restrictions. Findings suggest limited but consistent evidence that *A*. *Paniculata* improved cough and sore throat when compared with placebo. *A*. *Paniculata* (alone or plus usual care) has a statistically significant effect in improving overall symptoms of ARTIs when compared to placebo, usual care, and other herbal therapies. *A*. *Paniculata* in pillule tended to be more effective in improving overall symptoms over *A*. *Paniculata* in tablet. Evidence also suggested that *A*. *Paniculata* (alone or plus usual care) has shortened the duration of cough, sore throat and sick leave/time to resolution when compared versus usual care. Reduction in antibiotic usage was seldom evaluated in the included trials.

Although no serious AE was observed and minor AEs were mainly gastrointestinal in the included trials, caution is warranted in interpreting safety before comprehensive safety data is available. The quality of included trials was generally lower than desired as many were poorly designed, underpowered and inadequately blinded. There was high heterogeneity among trials due to variations in population and outcomes.

### Variations in *A*. *Paniculata*

#### Form of preparation and dosage

The two commonly prescribed preparations in the included trials were capsules and tablets; there were no decoctions. This may due to the extremely bitter nature of the herb described as the “king of bitters”. Findings of this review showed *A*. *Paniculata* pillules are superior to tablets in reliving overall symptoms [80–82], suggesting a place for pillule preparations.

Most *A*. *Paniculata* products have an extraction ratio of 14:1 standardised to contain an average of 35% of andrographolides [27] but solvent extraction ratios were not reported in most included trials. The amount of andrographolide produced from a daily dose of *A*. *Paniculata* extract varied from 15.75mg of andrographolide for URTIs [70], 225 mg for bronchiectasis [63], and up to 1200 mg for pharyngo-tonsillitis [52]. The most common treatment length was 5–7 days, ranging from 3 days for an AURTI [56] to 14 days for bronchiectasis [63] requiring administration three times daily. There is limited dose-finding research available documenting recommended percentage of active ingredient, dosage or ceiling effects so dosage is based in traditional use and herbal textbooks.

#### Common herbal combinations

The most commonly studied co-active ingredients included *Scutellaria baicalensis* (Huáng Qín [黄芩]) [50, 53–56, 58, 64–68, 71], *Isatidis Radix Isatidis* (Bǎn Lán Gēn [板蓝根]) [60, 61, 72–75], *Flos Lonicera* (Jīn Yín Huā [金银花]) [60, 61, 72–75], *Forsythia suspense* (Lián Qiào [连翘]) [60, 61, 72–74], and *Eleuthrococcus senticosus* (Cì Wǔ Jiā [刺五加]) [59, 69, 70, 79]. Apart from *Eleuthrococcus senticosus*, the other four herbs and *A*. *Paniculata* are commonly used heat-clearing anti-inflammatory and antimicrobial herbs in Traditional Chinese Medicine, along with *Coptis chinensis* (Huáng Lián [黄连]), *Folium* (Dà Qīng Yè [大青叶]), *Viola yedoensis* (Zî Huā Dì Dīng [紫花地丁]), *Pulsatilla Radix* (Bái Tóu Wēng [白头翁]), *Houttuynia cordata* (Yú Xīng Cǎo [鱼腥草]), and *Patrinia Herba* (Bài Jiàng Cǎo [败酱草]) [87]. Traditional Chinese Medicine (TCM) prescriptions often involve several herbs with synergistic actives which are frequently individualised based on the presenting symptoms and TCM diagnosis. This may result in complex phyto-pharmaceutical interactions and AEs.

#### Manufacturing

The review identified eight *A*. *Paniculata* products, representing four *A*. *Paniculata* polyherbal preparations (Ke Gan Shuang Qing^®^ capsule and tablet, Fu Fang Shuang Hua^®^ tablet and liquid, Kan Jang^®^ tablet, Jun Du Qing^®^ capsule) and four *A*. *Paniculata* monotherapies (Chuan Xin Lian Nei Zhi^®^ pillule and capsule, Chuan Xin Lian^®^ pillule, Kan Jang^®^ tablet, KalmCold^®^ capsule) (Table 6).

The active ingredients of *A*. *Paniculata* has not been fully identified in most trials but it is generally assumed to be the andrographolides. Only three trials [76–78] provided manufacturing details and chromatographic fingerprints of the herbal preparations to ensure quality and consistency of the products (Table 6). Those studies with inadequate information about the herbal content and manufacturing procedures may not be generalisable to other *A*. *Paniculata* studies as bioequivalence is ‘assumed’ rather than proven. A CONSORT herbal extension checklist is recommended to guide reporting of herbal trials and to assure herbal quality and bioequivalence.

### Safety (adverse events and toxicity)

The traditional uses of *A*. *Paniculata* are as a liver tonic to help maintain appetite and digestion; alleviate gastro-intestinal upsets and acute diarrhoea; immune function and to support intestinal function [27]. This traditional use may reduce adverse reactions caused by conventional medicines when they are prescribed in conjunction with *A Paniculata*. Findings of this review showed five cases of minor AEs in *A*. *Paniculata* group [71, 79, 81] (two cases were *A*. *Paniculata* plus usual care [64, 66]) and 48 cases [51, 58, 61, 64] were reported in control groups in the included trials. Minor AEs were mainly gastrointestinal, while there were two cases of dry mouth (Ribavirin [61]) and six cases of skin reaction (Cefixime [51] and *Echinacea purperea* [70]) reported. This was not consistent with the recent therapeutic goods administration (TGA) pharmacovigilance analysis, which revealed most common AEs associated with *A*. *Paniculata* were hypersensitivity or allergic reactions [29]. The TGA safety report explored association between anaphylactic/allergic type ADRs and *A*. *Paniculata*, suggesting that ADRs tend to be related to highly concentrated methanol extracts [29]. Our safety findings are inconclusive as there was an absence of proportionate data on each minor AE in each group thus limiting a comprehensive risk-benefit assessment.

Acute toxicity studies in rats suggested median lethal doses for andrograpolide is more than 40g/kg and 10 mg/kg body weight is when the ADRs became apparent [88]. The European Medicines Agency (EMA) reports no acute or genotoxicity data on Andrographis extracts but there is a possibilty of high doses causing reproductive toxicity, with decreases in sperm counts and motility that were linked to disruption of spermatogenesis in rats [89]. Animal research showed andrographolide-induced induction of CYP1A2, indicating an interaction with theophylline [90]. And Baicalin tends to interact with Omeprazole Chlorzoxazone Losartan [91], Rosuvastatin [92] and Acetaaminophen [93]. Mechanism of actions among herbal mixtures included in this review were not properly documented to support their use.

### Implications and future direction

This review suggests that *A*. *Paniculata* might act as a safe and effective treatment for ARTIs, either alone or in combination with usual care, as monotherapy or as a herbal mixture. Manufacturing information may be an important factor that differed among these included trials, and we recommend all further trials are based on a consistent, safe and well-defined *A*. *Paniculata* product. Pharmacological research exploring correlations between ADRs and manufacturing procedures (with methanol, or aqueous solvent, or aqueous-ethanol mixture) are also needed. Sensitivity analysis showed that higher quality trials suggested an enhanced improvement in overall symptoms and cough. Future well designed trials evaluating effectiveness and safety of oral *A*. *Paniculata* in capsule or tablet form and reported according to the herbal CONSORT checklist are vital and may serve to minise antibiotic prescription and AMR. The potential for antibiotic sparing should be studied in future trials.

### Strengths and limitations

Cochrane methodology was followed with a protocol of this systematic review registered and published online. A broad search strategy including both English and Chinese databases was adopted without language restrictions. Papers identified were screened and eligible trials extracted independently by two reviewers. We attempted to include grey literature by seeking manufacturers' reports and attempted to contact original authors for missing data. A number of studies including a substantial patient sample were identified; characteristics of the herb were documented following the criteria of CONSORT herbal extension.

Methodological quality of included trials was restricted as randomisation was not well documented; 73% of the trials included were not blinded; where ITT analysis were performed, loss to follow-up data were counted as no effect [56, 58, 73]; and most trials were published without a protocol available. The diagnostic criteria used in included trials were inconsistent and more than one third provided no inclusion/exclusion criteria. Not all trials were performed in countries where the International Council for Harmonisation (ICH) guidelines were legally binding. The included trials rarely clarified whether the products were GMP certified. However, methodological quality judgements were made on the basis of incomplete reporting the evidence of effectiveness may be undervalued [44]. Chinese-language randomised trials present a prominent excess of significant results that requires cautious interpretation [94]. It was not clear whether some of the trials were conducted with adequate ethical review; whether the products evaluated were not authenticated, or whether these details were poorly reported.

There were heterogeneities among trials included due to the heterogeneity population, clinical setting, variations in the form of *A*. *Paniculata* and controlled intervention employed, outcome measures, and different study protocols. Inadequate number of trials were available to allow further subgroup analyses on children or on lower ARTIs. Some included trials were non-inferiority RCTs as placebo control was considered unethical by some researchers. They demonstrated that *A*. *Paniculata* was clinically superior to other herbal interventions but failed to provide evidence on the established effect.

## Conclusions

*A*. *Paniculata* appears to be beneficial and safe for relieving ARTI symptoms and reducing time to symptom resolution. The evidence is inconclusive due to limited methodological quality of included trials and study heterogeneity. Well-designed trials evaluating effectiveness, efficacy and safety of *A*. *Paniculata* as a monotherapy, or as an herbal mixture, as well as exploring its potential to reduce antibiotic prescribing in primary care, are warranted.

## Supporting information

S1 Fig Risk of bias summary: Review authors' judgements about each risk of bias item for each included study. (TIF)

S1 Table PRISMA checklist. (DOC)

S2 Table Adverse events reported in the included trials. (DOCX)

S1 File PROSPERO international prospective register of systematic reviews. (DOC)

S2 File Search strategy of each database. (DOCX)
