# Correction: *Andrographis paniculata* (Chuān Xīn Lián) for symptomatic relief of acute respiratory tract infections in adults and children: A systematic review and meta-analysis

**DOI:** 10.1371/journal.pone.0207713

**Published:** 2018-11-14

**Authors:** Xiao-Yang Hu, Ruo-Han Wu, Martin Logue, Clara Blondel, Lily Yuen Wan Lai, Beth Stuart, Andrew Flower, Yu-Tong Fei, Michael Moore, Jonathan Shepherd, Jian-Ping Liu, George Lewith

The following information is missing from the Funding and Competing Interests section: ML’s PhD fellowship was supported by an educational stipend from Pukka Herbs.
