# Supplementary figures and images for: Andrographis paniculata (Chuān Xīn Lián) for symptomatic relief of acute respiratory tract infections in adults and children: A systematic review and meta-analysis
Source: PLoS One. 2017 Aug 4;12(8):e0181780. doi: 10.1371/journal.pone.0181780 (PMC5544222; doi:10.1371/journal.pone.0181780)

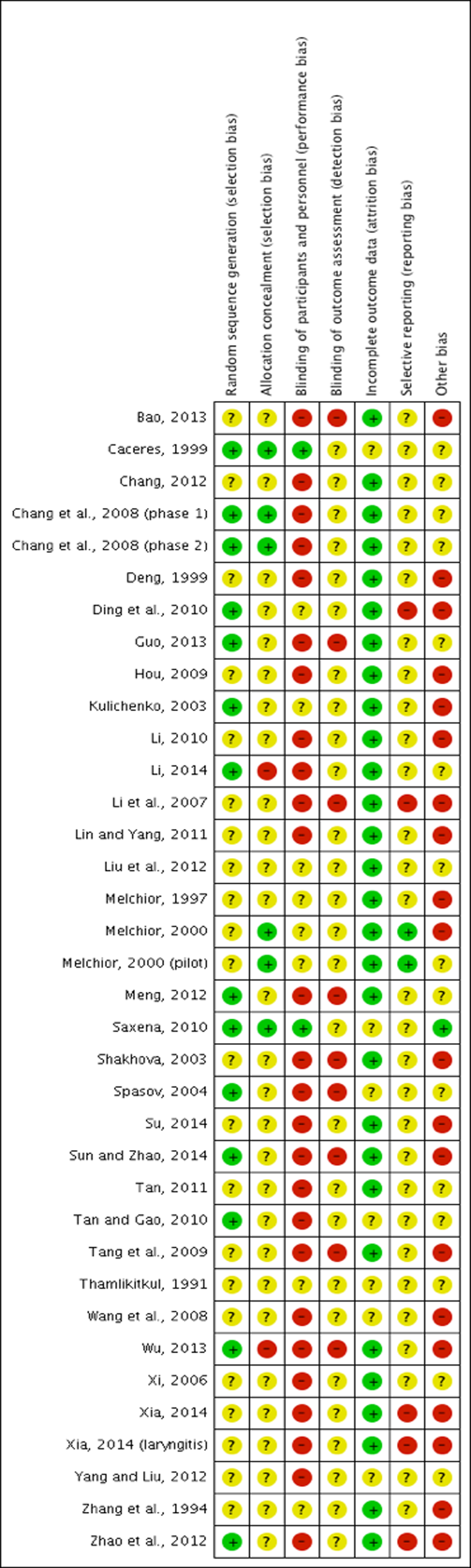

Supplement: S1 Fig — (TIF) [file pone.0181780.s001.tif]
