# Supplementary material for: Andrographis paniculata (Chuān Xīn Lián) for symptomatic relief of acute respiratory tract infections in adults and children: A systematic review and meta-analysis
Source: PLoS One. 2017 Aug 4;12(8):e0181780. doi: 10.1371/journal.pone.0181780 (PMC5544222; doi:10.1371/journal.pone.0181780)
Supplement: S2 Table — (DOCX) [file pone.0181780.s003.docx]

| AEs | *A. Paniculata* | Antibiotics | Antivirus | Other herbs |
| --- | --- | --- | --- | --- |
| Dry mouth |  |  | Ribavirin: 2 cases [63] |  |
| Constipation | Gan Ke Shuang Qing capsule: 1 case [68], 1 case [73] |  |  |  |
| “Gastrointestinal reactions” |  | Erythromycin ethylsuccinate: 19 cases [63] |  |  |
| Nausea | Andrographolide pillue: 1 case [82] | Cefixime: 2 cases [53] | Ribavirin: 11 cases [60] |  |
| Vomit | Ke Gan Shuang Qing capsule: 1 case [66] |  | Ribavirin: 2 cases [60], 2 cases [66] |  |
| Poor appetite |  |  | Ribavirin: 3 cases [66] |  |
| Rash |  | Cefixime: 5 cases [53] |  |  |
| Diarrhoea | Andrographolide tablet: 1 case [82] | Cefixime: 1 case [53] |  |  |
| Unpleasant sensations in the chest | Kan Jang tablet (with Eleuthrococcus senticosus): 1 case [81] |  |  |  |
| Intensified headache | Kan Jang tablet (with Eleuthrococcus senticosus): 1 case [81] |  |  |  |
| Allergic skin reaction |  |  |  | Immunal drop (Echinacea purperea): 1 case [72] |

N.B. Some AEs cannot be identified in this table are reported in text
