# Supplementary material for: Andrographis paniculata (Chuān Xīn Lián) for symptomatic relief of acute respiratory tract infections in adults and children: A systematic review and meta-analysis
Source: PLoS One. 2017 Aug 4;12(8):e0181780. doi: 10.1371/journal.pone.0181780 (PMC5544222; doi:10.1371/journal.pone.0181780)
Supplement: S2 File — (DOCX) [file pone.0181780.s005.docx]

**MEDLINE (Ovid): From 1946 to March 2016**

1. exp Respiratory Tract Infections/

2. (respiratory tract infection* or (respiratory adj3 infection*) or RTI* or (chest adj3 infection*) or upper respiratory tract infection* or upper respiratory infection* or lower respiratory tract infection* or lower respiratory infection*).mp.

3. exp Rhinitis/

4. exp Sinusitis/

5. exp Pharyngitis/

6. Nasopharyngitis/

7. exp Laryngitis/

8. (rhinit* or sinusit* or pharyngit* or laryngit* or rhinosinusit* or rhinopharyngit* or rhinolaryngit* or nasosinusit* or nasopharyngit* or nasolaryngit* or sinonasal* or

rhino-sinusit* or rhino-pharyngit* or rhino-laryngit* or naso-sinusit* or naso-pharyngit* or sino-nasal*).mp.

9. exp Bronchitis/

10. exp Supraglottitis/

11. Tracheitis/

12. exp Pneumonia/

13. (bronchit* or supraglottit* or epiglott* or peumon* or pulmon* or tracheit* or brochopneumon* or pleuropneumon* or respirat*).mp.

14. (cough* or cold* or catarrh or flu or influenza or (sore adj3 throat) or (throat adj3 pain) or (blocked adj3 nose) or (runn* adj3 nose) or (stuff* adj3 nose) or (short* adj3 breath*) or rhinorrh?ea or congest* or discharge*).mp.

15. or/1-14

16. Andrographis/

17. (andrograph* or paniculata or andrographis paniculata or king of bitter*

or kalmegh* or kalamegh* or nilavembu or nila-vembu or kanjang or kan-jang or kiryat or chiretta or fa-ta-lai-jone or fa-talai-jone or chuanxinlian or chuan-xin-lian or yijianxi or yi-jian-xi or lanhelian or lan-he-lian or Indian-echinacea or immunoguard or livfit or livo-plus or didehydroandrographolide or didehydro-andrographolide or dehydroandrographolide or dehydro-andrographolide or neoandrographolide or neo-andrographolide or andrograpanin).mp.

18. 16 or 17

19. 15 and 18

Limit 19 to human(s)

*=truncation, exp=explode, adj3=adjacent within 3 words of each other in either direction, ?=substitute for one or no characters

mp=title, abstract, original title, name of substance word, subject heading word, keyword heading word, protocol supplementary concept word, rare disease supplementary concept word, unique identifier

**AMED (Ovid)**: **From 1985 to March 2016**

1. exp Respiratory Tract Infections/

2. (respiratory tract infection* or (respiratory adj3 infection*) or RTI* or (chest adj3 infection*) or upper respiratory tract infection* or upper respiratory infection* or lower respiratory tract infection* or lower respiratory infection*).mp.

3. exp Rhinitis/

4. exp Sinusitis/

5. exp Pharyngitis/

6. Nasopharyngitis/

7. (rhinit* or sinusit* or pharyngit* or laryngit* or rhinosinusit* or rhinopharyngit* or rhinolaryngit* or nasosinusit* or nasopharyngit* or nasolaryngit* or sinonasal* or

rhino-sinusit* or rhino-pharyngit* or rhino-laryngit* or naso-sinusit* or naso-pharyngit* or sino-nasal*).mp.

8. exp Bronchitis/

9. exp Supraglottitis/

10. Tracheitis/

11. exp Pneumonia/

12. (bronchit* or supraglottit* or epiglott* or peumon* or pulmon* or tracheit* or brochopneumon* or pleuropneumon* or respirat*).mp.

13. (cough* or cold* or catarrh or flu or influenza or (sore adj3 throat) or (throat adj3 pain) or (blocked adj3 nose) or (runn* adj3 nose) or (stuff* adj3 nose) or (short* adj3 breath*) or rhinorrh?ea or congest* or discharge*).mp.

14. or/1-13

15. (andrograph* or paniculata or andrographis paniculata or king of bitter*

or kalmegh* or kalamegh* or nilavembu or nila-vembu or kanjang or kan-jang or kiryat or chiretta or fa-ta-lai-jone or fa-talai-jone or chuanxinlian or chuan-xin-lian or yijianxi or yi-jian-xi or lanhelian or lan-he-lian or Indian-echinacea or immunoguard or livfit or livo-plus or didehydroandrographolide or didehydro-andrographolide or dehydroandrographolide or dehydro-andrographolide or neoandrographolide or neo-andrographolide or andrograpanin).mp.

16. 14 and 15

*=truncation, exp=explode, adj3=adjacent within 3 words of each other in either direction, ?=substitute for one or no characters

mp=title, abstract, original title, name of substance word, subject heading word, keyword heading word, protocol supplementary concept word, rare disease supplementary concept word, unique identifier

**Embase (Ovid): From 1947 to March 2016**

1. exp Respiratory Tract Infections/

2. (respiratory tract infection* or (respiratory adj3 infection*) or RTI or (chest adj3 infection*) or upper respiratory tract infection* or upper respiratory infection* or lower respiratory tract infection* or lower respiratory infection*).mp.

3. exp Rhinitis/

4. exp Sinusitis/

5. exp Pharyngitis/

6. Nasopharyngitis/

7. exp Laryngitis/

8. (rhinit* or sinusit* or pharyngit* or laryngit* or rhinosinusit* or rhinopharyngit* or rhinolaryngit* or nasosinusit* or nasopharyngit* or nasolaryngit* or sinonasal* or

rhino-sinusit* or rhino-pharyngit* or rhino-laryngit* or naso-sinusit* or naso-pharyngit* or sino-nasal*).mp.

9. exp Bronchitis/

10. exp Supraglottitis/

11. Tracheitis/

12. exp Pneumonia/

13. (bronchit* or supraglottit* or epiglott* or peumon* or pulmon* or tracheit* or brochopneumon* or pleuropneumon* or respirat*).mp.

14. (cough* or cold* or catarrh or flu or influenza or (sore adj3 throat) or (throat adj3 pain) or (blocked adj3 nose) or (runn* adj3 nose) or (stuff* adj3 nose) or (short* adj3 breath*) or rhinorrh?ea or congest* or discharge*).mp.

15. or/1-14

16. Andrographis/

17. (andrograph* or paniculata or andrographis paniculata or king of bitter*

or kalmegh* or kalamegh* or nilavembu or nila-vembu or kanjang or kan-jang or kiryat or chiretta or fa-ta-lai-jone or fa-talai-jone or chuanxinlian or chuan-xin-lian or yijianxi or yi-jian-xi or lanhelian or lan-he-lian or Indian-echinacea or immunoguard or livfit or livo-plus or didehydroandrographolide or didehydro-andrographolide or dehydroandrographolide or dehydro-andrographolide or neoandrographolide or neo-andrographolide or andrograpanin).mp.

18. 16 or 17

19. 15 and 18

Limit 19 to human(s)

*=truncation, exp=explode, adj3=adjacent within 3 words of each other in either direction, ?=substitute for one or no characters

mp=title, abstract, original title, name of substance word, subject heading word, keyword heading word, protocol supplementary concept word, rare disease supplementary concept word, unique identifier

**CINAHL Plus with Full Text (EBSCO): From 1937 to March 2016**

1. (MH "Respiratory Tract Infections+")

2. TI respiratory tract infection* or AB respiratory tract infection* or SU respiratory tract infection* or TI (respiratory N3 infection*) or AB (respiratory N3 infection*) or SU (respiratory N3 infection*) or TI RTI* or AB RTI* or SU RTI* or TI (chest N3 infection*) or AB (chest N3 infection*) or SU (chest N3 infection*)

3. TI (rhinit* or sinusit* or pharyngit* or laryngit* or rhinosinusit* or rhinopharyngit* or rhinolaryngit* or nasosinusit* or nasopharyngit* or nasolaryngit* or sinonasal* or rhino-sinusit* or rhino-pharyngit* or rhino-laryngit* or naso-sinusit* or naso-pharyngit* or sino-nasal*) or AB (rhinit* or sinusit* or pharyngit* or laryngit* or rhinosinusit* or rhinopharyngit* or rhinolaryngit* or nasosinusit* or nasopharyngit* or nasolaryngit* or sinonasal* or rhino-sinusit* or rhino-pharyngit* or rhino-laryngit* or naso-sinusit* or naso-pharyngit* or sino-nasal*) or SU (rhinit* or sinusit* or pharyngit* or laryngit* or rhinosinusit* or rhinopharyngit* or rhinolaryngit* or nasosinusit* or nasopharyngit* or nasolaryngit* or sinonasal* or rhino-sinusit* or rhino-pharyngit* or rhino-laryngit* or naso-sinusit* or naso-pharyngit* or sino-nasal*)

4. TI (bronchit* or supraglottit* or epiglott* or peumon* or pulmon* or tracheit* or brochopneumon* or pleuropneumon* or respirat*) or AB (bronchit* or supraglottit* or epiglott* or peumon* or pulmon* or tracheit* or brochopneumon* or pleuropneumon* or respirat*) or SU (bronchit* or supraglottit* or epiglott* or peumon* or pulmon* or tracheit* or brochopneumon* or pleuropneumon* or respirat*)

5. TI (cough* or cold* or catarrh or flu or influenza or (sore adj3 throat) or (throat adj3 pain) or (blocked adj3 nose) or (runn* adj3 nose) or (stuff* adj3 nose) or (short* adj3 breath*) or rhinorrh?ea or congest* or discharge*) or AB (cough* or cold* or catarrh or flu or influenza or (sore adj3 throat) or (throat adj3 pain) or (blocked adj3 nose) or (runn* adj3 nose) or (stuff* adj3 nose) or (short* adj3 breath*) or rhinorrh?ea or congest* or discharge*) or SU (cough* or cold* or catarrh or flu or influenza or (sore adj3 throat) or (throat adj3 pain) or (blocked adj3 nose) or (runn* adj3 nose) or (stuff* adj3 nose) or (short* adj3 breath*) or rhinorrh?ea or congest* or discharge*)

6. TI ((cough* or cold* or catarrh or flu or influenza or (sore N3 throat) or (throat N3 pain) or (blocked N3 nose) or (runn* N3 nose) or (stuff* N3 nose) or (short* N3 breath*) or rhinorrh?ea or congest* or discharge*)) or AB ((cough* or cold* or catarrh or flu or influenza or (sore N3 throat) or (throat N3 pain) or (blocked N3 nose) or (runn* N3 nose) or (stuff* N3 nose) or (short* N3 breath*) or rhinorrh?ea or congest* or discharge*)) or SU ((cough* or cold* or catarrh or flu or influenza or (sore N3 throat) or (throat N3 pain) or (blocked N3 nose) or (runn* N3 nose) or (stuff* N3 nose) or (short* N3 breath*) or rhinorrh?ea or congest* or discharge*))

7. or/1-6

8. TI (andrograph* or paniculata or andrographis paniculata or king of bitter* or kalmegh* or kalamegh* or nilavembu or nila-vembu or kanjang or kan-jang or kiryat or chiretta or fa-ta-lai-jone or fa-talai-jone or chuanxinlian or chuan-xin-lian or yijianxi or yi-jian-xi or lanhelian or lan-he-lian or Indian-echinacea or immunoguard or livfit or livo-plus or didehydroandrographolide or didehydro-andrographolide or dehydroandrographolide or dehydro-andrographolide or neoandrographolide or neo-andrographolide or andrograpanin) or AB (andrograph* or paniculata or andrographis paniculata or king of bitter* or kalmegh* or kalamegh* or nilavembu or nila-vembu or kanjang or kan-jang or kiryat or chiretta or fa-ta-lai-jone or fa-talai-jone or chuanxinlian or chuan-xin-lian or yijianxi or yi-jian-xi or lanhelian or lan-he-lian or Indian-echinacea or immunoguard or livfit or livo-plus or didehydroandrographolide or didehydro-andrographolide or dehydroandrographolide or dehydro-andrographolide or neoandrographolide or neo-andrographolide or andrograpanin) or SU (andrograph* or paniculata or andrographis paniculata or king of bitter* or kalmegh* or kalamegh* or nilavembu or nila-vembu or kanjang or kan-jang or kiryat or chiretta or fa-ta-lai-jone or fa-talai-jone or chuanxinlian or chuan-xin-lian or yijianxi or yi-jian-xi or lanhelian or lan-he-lian or Indian-echinacea or immunoguard or livfit or livo-plus or didehydroandrographolide or didehydro-andrographolide or dehydroandrographolide or dehydro-andrographolide or neoandrographolide or neo-andrographolide or andrograpanin)

9. 7 and 8

*=truncation, N3=finds the words if they are within five words of one another, regardless of the order in which they appear, ?= replaces that number of character(s)

MH=MeSH, TI=title, AB=abstract, SU=subject,

**Cochrane Central Register of Controlled Trials (CENTRAL) on the Cochrane Library: From inception to March 2016**

1. MeSH descriptor: [Respiratory Tract Infections] explode all trees

2. (respiratory tract infection* or (respiratory near infection*) or RTI or (chest near infection*) or upper respiratory tract infection* or upper respiratory infection* or lower respiratory tract infection* or lower respiratory infection*)

3. (rhinit* or sinusit* or pharyngit* or laryngit* or rhinosinusit* or rhinopharyngit* or rhinolaryngit* or nasosinusit* or nasopharyngit* or nasolaryngit* or sinonasal* or

rhino-sinusit* or rhino-pharyngit* or rhino-laryngit* or naso-sinusit* or naso-pharyngit* or sino-nasal*)

4. (bronchit* or supraglottit* or epiglott* or peumon* or pulmon* or tracheit* or brochopneumon* or pleuropneumon* or respirat*)

5. (cough* or cold* or catarrh or flu or influenza or (sore near throat) or (throat near pain) or (blocked adj3 nose) or (runn* adj3 nose) or (stuff* near nose) or (short* near breath*) or rhinorrh?ea or congest* or discharge*)

6. #1 or #2 or #3 or #4 or #5

7. MeSH descriptor: [Andrographis] explode all trees

8. (andrograph* or paniculata or andrographis paniculata or king of bitter*

or kalmegh* or kalamegh* or nilavembu or nila-vembu or kanjang or kan-jang or kiryat or chiretta or fa-ta-lai-jone or fa-talai-jone or chuanxinlian or chuan-xin-lian or yijianxi or yi-jian-xi or lanhelian or lan-he-lian or Indian-echinacea or immunoguard or livfit or livo-plus or didehydroandrographolide or didehydro-andrographolide or dehydroandrographolide or dehydro-andrographolide or neoandrographolide or neo-andrographolide or andrograpanin)

9. #7 or #8

10. #6 and #9

19. #15 AND #18

*=truncation, exp=explode, adj3=adjacent within 3 words of each other in either direction, ?=substitute for one or no characters

mp=title, abstract, original title, name of substance word, subject heading word, keyword heading word, protocol supplementary concept word, rare disease supplementary concept word, unique identifier

**China Network Knowledge Infrastructure (CNKI): From inception to March 2016**

SU=('呼吸道感染'+'鼻炎'+'鼻窦炎'+'咽炎'+'喉炎'+'鼻咽炎'+'扁桃体炎'+'支气管炎'+'气管炎'+'肺炎'+'咳嗽'+'感冒'+'外感'+'流感'+'喉咙痛'+'咽痛'+'咽喉痛'+'咽痒'+'鼻塞'+'流涕'+'咳'+'嗽'+'喘'+'肺'+'鼻'+'咽'+'喉') AND SU=('穿心莲'+'圆锥药须草'+'槛核莲'+'一见喜'+'斩舌剑'+'苦草'+'苦胆草'+'四方草'+'斩蛇剑'+'日行千里感'+'四方莲'+'金香草'+'金耳钩'+'春莲夏柳'+'印度草'+'万病仙草'+'四支邦'+'斩龙剑'+'春莲秋柳'+'清感双舒'+'复方双花'+'感咳双清'+'新雪丹'+'喉康散'+'感冒清')

SU= subject heading

**Chinese Scientific Journals Database (VIP): From inception to March 2016**

M=(呼吸道感染+鼻炎+鼻窦炎+咽炎+喉炎+鼻咽炎+扁桃体炎+支气管炎+气管炎+肺炎+咳嗽+感冒+外感+流感+喉咙痛+咽痛+咽喉痛+咽痒+鼻塞+流涕+咳+嗽+喘+肺+鼻+咽+喉) * M=(穿心莲+圆锥药须草+槛核莲+一见喜+斩舌剑+苦草+苦胆草+四方草+斩蛇剑+日行千里感+四方莲+金香草+金耳钩+春莲夏柳+印度草+万病仙草+四支邦+斩龙剑+春莲秋柳+清感双舒+复方双花+感咳双清+新雪丹+喉康散+感冒清)

M= title and abstract

**Wan Fang database: From inception to March 2016**

主题:('呼吸道感染'+'鼻炎'+'鼻窦炎'+'咽炎'+'喉炎'+'鼻咽炎'+'扁桃体炎'+'支气管炎'+'气管炎'+'肺炎'+'咳嗽'+'感冒'+'外感'+'流感'+'喉咙痛'+'咽痛'+'咽喉痛'+'咽痒'+'鼻塞'+'流涕'+'咳'+'嗽'+'喘'+'肺'+'鼻'+'咽'+'喉') * 主题:('穿心莲'+'圆锥药须草'+'槛核莲'+'一见喜'+'斩舌剑'+'苦草'+'苦胆草'+'四方草'+'斩蛇剑'+'日行千里感'+'四方莲'+'金香草'+'金耳钩'+'春莲夏柳'+'印度草'+'万病仙草'+'四支邦'+'斩龙剑'+'春莲秋柳'+'清感双舒'+'复方双花'+'感咳双清'+'新雪丹'+'喉康散'+'感冒清')

主题= subject heading

**Sino-Med Database: From inception to March 2016**

1. ((((((("呼吸道感染"[中文标题:智能]) OR "呼吸道感染"[摘要:智能]) OR "鼻炎"[中文标题:智能]) OR "鼻炎"[摘要:智能]) OR "鼻窦炎"[中文标题:智能]) OR "鼻窦炎"[摘要:智能]) OR "咽炎"[中文标题:智能]) OR "咽炎"[摘要:智能]

2. ((((((("喉炎"[中文标题:智能]) OR "喉炎"[摘要:智能]) OR "鼻咽炎"[中文标题:智能]) OR "鼻咽炎"[摘要:智能]) OR "扁桃体炎"[中文标题:智能]) OR "扁桃体炎"[摘要:智能]) OR "支气管炎"[中文标题:智能]) OR "支气管炎"[摘要:智能]

3. ((((((("气管炎"[中文标题:智能]) OR "气管炎"[摘要:智能]) OR "肺炎"[中文标题:智能]) OR "肺炎"[摘要:智能]) OR "咳嗽"[中文标题:智能]) OR "咳嗽"[摘要:智能]) OR "感冒"[中文标题:智能]) OR "感冒"[摘要:智能]

4. ((((((("外感"[中文标题:智能]) OR "外感"[摘要:智能]) OR "流感"[中文标题:智能]) OR "流感"[摘要:智能]) OR "喉咙痛"[中文标题:智能]) OR "喉咙痛"[摘要:智能]) OR "咽痛"[中文标题:智能]) OR "咽痛"[摘要:智能]

5. ((((((("咽喉痛"[中文标题:智能]) OR "咽喉痛"[摘要:智能]) OR "咽痒"[中文标题:智能]) OR "咽痒"[摘要:智能]) OR "鼻塞"[中文标题:智能]) OR "鼻塞"[摘要:智能]) OR "流涕"[中文标题:智能]) OR "流涕"[摘要:智能]

6. ((((((((((((("咳"[中文标题:智能]) OR "咳"[摘要:智能]) OR "嗽"[中文标题:智能]) OR "嗽"[摘要:智能]) OR "喘"[中文标题:智能]) OR "喘"[摘要:智能]) OR "肺"[中文标题:智能]) OR "肺"[摘要:智能]) OR "鼻"[中文标题:智能]) OR "鼻"[摘要:智能]) OR "咽"[中文标题:智能]) OR "咽"[摘要:智能]) OR "喉"[中文标题:智能]) OR "喉"[摘要:智能]

7. 1+2+3+4+5+6

8. ((((((((("穿心莲"[中文标题:智能]) OR "穿心莲"[摘要:智能]) OR "圆锥药须草"[中文标题:智能]) OR "圆锥药须草"[摘要:智能]) OR "槛核莲"[中文标题:智能]) OR "槛核莲"[摘要:智能]) OR "一见喜"[中文标题:智能]) OR "一见喜"[摘要:智能]) OR "斩舌剑"[中文标题:智能]) OR "斩舌剑"[摘要:智能]

9. ((((((((("苦草"[中文标题:智能]) OR "苦草"[摘要:智能]) OR "苦胆草"[中文标题:智能]) OR "苦胆草"[摘要:智能]) OR "四方草"[中文标题:智能]) OR "四方草"[摘要:智能]) OR "斩蛇剑"[中文标题:智能]) OR "斩蛇剑"[摘要:智能]) OR "日行千里感"[中文标题:智能]) OR "日行千里感"[摘要:智能]

((((((((("四方莲"[中文标题:智能]) OR "四方莲"[摘要:智能]) OR "金香草"[中文标题:智能]) OR "金香草"[摘要:智能]) OR "金耳钩"[中文标题:智能]) OR "金耳钩"[摘要:智能]) OR "春莲夏柳"[中文标题:智能]) OR "春莲夏柳"[摘要:智能]) OR "印度草"[中文标题:智能]) OR "印度草"[摘要:智能]

((((((((("万病仙草"[中文标题:智能]) OR "万病仙草"[摘要:智能]) OR "四支邦"[中文标题:智能]) OR "四支邦"[摘要:智能]) OR "斩龙剑"[中文标题:智能]) OR "斩龙剑"[摘要:智能]) OR "春莲秋柳"[中文标题:智能]) OR "春莲秋柳"[摘要:智能]) OR "清感双舒"[中文标题:智能]) OR "清感双舒"[摘要:智能]

((((((((("复方双花"[中文标题:智能]) OR "复方双花"[摘要:智能]) OR "感咳双清"[中文标题:智能]) OR "感咳双清"[摘要:智能]) OR "新雪丹"[中文标题:智能]) OR "新雪丹"[摘要:智能]) OR "喉康散"[中文标题:智能]) OR "喉康散"[摘要:智能]) OR "感冒清"[中文标题:智能]) OR "感冒清"[摘要:智能]

10. 8+9

11. 7*10
